# Toward a cell-type-specific lung HOX code

**DOI:** 10.3389/fcell.2026.1839422

**Published:** 2026-07-14

**Authors:** Bettina Budeus, Diana Klein

**Affiliations:** Medical Faculty, Institute for Cell Biology (Cancer Research), University of Duisburg-Essen, Essen, Germany

**Keywords:** alveolar, bronchiolar, epithelia cells, fibroblasts, HOX code, lung, organoids

## Abstract

**Background:**

HOX genes encode transcription factors that are central regulators of cell fate during embryogenesis and of maintaining respective positional cell identity throughout life. Moreover, HOX factors shape the specification of regional properties of the respective tissues, but detailed information regarding the role of HOX genes in the adult human respiratory system is still lacking.

**Methods:**

We examined the HOX expression pattern in human lungs using global gene expression analyses of normal lung tissue specimens, followed by quantitative real-time reverse-transcriptase polymerase chain reaction (qRT-PCR). Cell-type specificity was investigated using published single-cell RNA sequencing (scRNAseq) datasets and by immunohistochemistry and immunofluorescence analyses.

**Results:**

The expressed HOX candidates HOXA3–HOXA7, HOXB5, HOXB7, and HOXB8, HOXC6–HOXC9, and HOXD1–D8 were localized in healthy lung specimens. Early and central HOXA and HOXB genes were found to be more highly expressed in adult lung tissue, predominantly localizing in stromal cells. While HOXA3 and HOXA5 were primarily localized in sub-bronchial and vascular smooth muscle cells (SMCs), HOXA4, HOXB5, and HOXB7 were predominantly found in vascular mural cells, whereas expression in sub-bronchial regions was more associated with connective tissue cells. HOXA6 was exclusively detected in sub-bronchial and adventitial connective tissue; a similar pattern was observed for HOXA7. HOXA7, HOXB5, and HOXB7, along with HOXD1, were found in the endothelial cells of predominantly larger vessels. Bronchial epithelial cells showed immunoreactivity for almost all the HOX proteins examined, although in some cases, this was limited to single cells. In the alveolar compartment, only HOXA6 (presumably in connective tissue cells), as well as HOXB6, HOXC6, and HOXC8, could be localized. A temporal analysis of the HOX expression development during lung organoid differentiation from induced pluripotent stem cells confirmed respective findings.

**Conclusion:**

Since the deregulation of HOX genes is increasingly observed in numerous (malignant) lung diseases, it is a necessary prerequisite to specify individual HOX factors and their cellular origin. This could not only provide better insights into the function of HOX genes in lung diseases but also offer the possibility of improving the regenerative potential through targeted modulation of (cell-type-specific) HOX genes.

## Background

HOX genes are homeobox-containing, protein-coding genes arranged in four chromosomal clusters, with the genomic arrangement reflecting sequential activation during development, where genes at the 3′ end are expressed earlier in anterior regions, while genes at the 5′ end are activated later in posterior regions (Sikkema et al., 2023; Dasen, 2013). The respective HOX proteins are transcription factors that function as master regulators of development and control the specification of all cell types and tissues (Pearson et al., 2005; Pinto et al., 2024). The overlapping but still different HOX gene expression patterns found here suggested a combinatorial code of HOX gene expressions that determines the subsequent development of certain body regions (Kappen, 1996). Although some HOX-dependent regulatory signaling networks have been characterized and thus gained insight how HOX proteins regulate downstream targets to mediate the formation of segment-specific structures, detailed mechanisms of HOX-controlled cellular functions remain elusive (Rezsohazy et al., 2015; Hubert and Wellik, 2023). Developmentally, critical roles for certain HOX genes have been demonstrated in lung organogenesis. Based on studies in mice, it is generally assumed that Hox genes of paralogous groups 3–6 contribute directly to lung development by regulating the expression of lung-specific transcription factors, thereby ultimately leading to the differentiation of specific lung regions (Kappen, 1996). Loss-of-function studies revealed a developmental need of Hoxa1 and Hoxa3 for proper pulmonary function and respiration (Lufkin et al., 1991; Chisaka and Capecchi, 1991). Hoxa3, furthermore, was found to be important for proper specification of tissues in the tracheal region (Kappen, 1996; Duboule and Morata, 1994). It was later shown that Hox genes of the paralog groups 3 and 7 in clusters A and B were the most strongly represented in the mouse lung, with Hoxa4 and Hoxa5 being the most highly transcribed (Grier et al., 2009). Although HoxA4 was highly transcribed during embryogenesis (at E 14.5), the consistently high transcription of Hoxa5 (up to postnatal day 20) was supposed to implicate a role for Hoxa5 in normal lung maturation (Grier et al., 2009). Steady-state mRNA levels of Hoxa5, however, together with Hoxb5, Hoxb6, and Hoxb8, were shown to decrease in murine lungs upon lung development (from E14 to adult) (Bogue et al., 1994). Hoxa5 was mainly localized to the mesodermal compartment. A genetic deficiency of Hoxa5 resulted perinatal lethality due to improper tracheal and lung morphogenesis, effects that were accompanied by a disorganization of the mesenchymal layer surrounding secondary bronchi, bronchioles, and terminal acini that were even reduced in numbers (Aubin et al., 1997). Similarly, Hoxa4 was clearly implicated in mouse lung development and patterning, with Hoxa4 expressions increasing in the mesenchymal compartment of the lung (at E 12.5) (Packer et al., 2000). Later on, HOXA4, together with HOXA5 expression, was found in the proximal mesenchyme and in smooth muscle cells (SMCs), subepithelial fibroblasts, and alveolar epithelial cells (Packer et al., 2000). Nowadays, HOXA5 is known to regulate reciprocal interactions between the epithelium and the mesenchyme, being decisive for controlling lung development, particularly epithelial differentiation and branching (Landry-Truchon et al., 2017). Using mutant mice, deficiency of Hox5 (Hoxa5, Hoxb5, and Hoxc5) genes altogether highlighted the need of these HOX genes not only for branching morphogenesis but also for goblet cell specification and postnatal air space structure, particularly for the generation of the elastin network by lung fibroblasts (Hrycaj et al., 2018), which, in turn, is required for alveolar formation (Li et al., 2021).

Thus, it is convincing that HOX genes decisively regulate branching morphogenesis, epithelial cell fate, and the differentiation of conducting airway epithelium during murine lung embryogenesis and development. Overall, changes in Hox gene expression, as observed in mutant mice, primarily affected mesodermal derivatives, which, in turn, act upon the epithelium (Kappen, 1996; Goodwin et al., 2022; Golpon et al., 2001). However, most studies have focused on HOX gene expression during embryonic development in the mouse; consequently, little information is available regarding their expression in adult tissues, particularly in the adult human lung. In adult organisms, cells can retain the characteristic Hox expression patterns that prevailed during development—a phenomenon understood as a cell- and/or tissue-specific “Hox code” that reflects the positional identity of the cells (Steens and Klein, 2022; Mann and Glassford, 2024; Deschamps et al., 2017; Kmita and Duboule, 2003). In a first, very elegant study, the expression pattern of HOX genes was investigated in different human lung tissue specimens (Golpon et al., 2001). Using microarray and degenerate reverse-transcriptase polymerase chain reaction (RT-PCR) survey techniques, it was revealed that HOX genes predominantly from the anterior region of clusters A and B were expressed in normal lungs. Quantification of HOX gene expression in human fetal lungs compared to adult lungs identified HOXA5 as the most abundantly expressed HOX gene, followed by HOXB2 and HOXB6, in both tissue samples, although respective overall expression levels of HOX candidates in adult lung tissues were lower (Golpon et al., 2001). Posterior HOX genes paralogs (HOX10 to HOX13) were not detected. Of note, under diseased conditions (in emphysema and primary pulmonary hypertension lung specimen), HOX genes from clusters C and D were found to be additionally expressed (Golpon et al., 2001). Differences in HOX gene expression between normal and diseased lungs increasingly suggest that HOX genes may play central roles in promoting pulmonary diseases, e.g., fibrosis and cancer (Li et al., 2019; Clemenceau et al., 2018; Calvo et al., 2000; Samarell et al., 2024; Li et al., 2021). Therefore, there is an urgent need to better understand the biology of HOX family members in the adult system and to further elucidate corresponding gene targets and molecular networks. The aim of this study was to determine HOX-specific expression patterns in adult lung tissue—thereby defining a potential lung HOX code—and to ascertain the potential cell-type specificity of this expression pattern.

## Methods

### Histology and immunofluorescence

Immunohistochemistry (IHC) and immunofluorescence staining were performed on formalin-fixed and paraffin-embedded lung tissue as previously described (Ketteler et al., 2020; Steens et al., 2021). Normal lung tissue samples were obtained from surgical resections according to local ethical and biohazard regulations. Concerning the anatomical regions, lung tissues mainly originated from medial and distal (alveolar) lung regions. Written informed consent (17-7454-BO) was obtained from the Ethikkommission of the University Medical Faculty, Essen, Germany. Samples were collected in close cooperation with the local biobank (Westdeutsche Biobank Essen) and were analyzed anonymously. In brief, paraffin-embedded tissue sections were hydrated using a descending alcohol series, incubated for 10–20 min in target retrieval solution (DAKO, Glostrup, Denmark). The sections were incubated with primary antibodies in blocking solution (2% FCS/PBS) overnight at 4 °C. Antigens were detected with respective fluorescence-conjugated secondary antibodies (1/500) in combination with Hoechst 33342 nucleic acid stain or with horseradish peroxidase-conjugated secondary antibodies (1/500) and DAB staining (Ketteler et al., 2020; Steens et al., 2021). The following antibodies were used: CD34 (Clone QBEnd; #10716501-2) was purchased from DAKO/Agilent Technologies (Santa Clara, CA). SMA/ACTA2 (B4; #sc-53142), HOXA5 (C-11; #sc365784), HOXB5 (133C3a; #sc81099), vimentin (RV202; #sc32322), and CD68 (KP1; #sc20060) were purchased from Santa Cruz Biotechnology, Inc (Dallas, TX). Acetyl-alpha tubulin (Lys40) (6-11B-1; # 32-2700), HOXA3 (#PA5-26887), HOXA4 (#PA5—79385), HOXB6 (#PA5-116164), HOXB8 (#BS-6539R), HOXC5 (#BS-11589R), HOXC6 (#PA5-41479), HOXC9 (#PA5-67618), HOXD1 (# PA5-113456), HOXD4 (OTI1E9; #MA5-27158), and HOXD8 (#PA5-27377) antibodies were obtained from ThermoFischer Scientific (Waltham, MA). HOXA5 (#27622-1-AP), HOXA6 (#18210-1-AP), HOXA7 (#12613-1-Ig), HOXB5, HOXB7 (#12613-1-1-AP), HOXC8 (# 15448-1-AP), ACTA2/SMA (#67735-1-Ig), CAVEOLIN-1 (#16447-1-AP), transgelin/SM22 (#10493-1-AP), and TTF1/NKX2-1 (#66034-1-Ig) antibodies were obtained from Proteintech Group, Inc. (Rosemont, IL).

### Generation and culture of lung organoids

Lung organoids were generated from induced pluripotent stem cells (iPSCs) as previously described (Budeus et al., 2024; Budeus et al., 2025). In brief, human iPSCs (#SCTi003; StemCell Technologies, Vancouver, BC, Canada; #iPS01, ALSTEM Inc., Richmond, CA, United States) were maintained on Vitronectin XF-coated culture dishes with mTeSR Plus medium (StemCell Technologies) under standard cell culture conditions at 37 °C and 5% CO_2_. Cells were routinely tested for *mycoplasma* contamination (every month). For organoid generation, iPSCs were harvested using an enzyme-free cell dissociation reagent (GCDR, #100-0485; StemCell Technologies), counted, and seeded into ultra-low attachment BIOFLOAT 96-well plates (#83.3925.400; SARSTEDT, Nümbrecht, Germany) in mTeSR Plus medium with 2,500 cells per 100 µL per well. After 4–5 days, 100 µL per well of BLO-medium was added to generated embryoid bodies. After additional 4–5 days, a complete media change was performed, and structures were cultured for 28–52 days with complete media changes every other day. The BLO medium consists of DMEM F12 with GlutaMAX™ Supplement (#10565018; GIBCO/Invitrogen/Thermo Fisher Scientific, Carlsbad, CA, United States), 1× N-2, 1× B27, 1× penicillin–streptomycin (5,000 U/mL), 0.4% (vol/vol) BSA, 0.4 µM monothioglycerol, 50 μg/mL ascorbic acid, 10 ng/mL KGF/FGF7, 10 ng/mL FGF10, 50 nM ATRA, and 3 µM CHIR-99021.

### RNA sequencing analysis

Total RNA was isolated from normal lung tissue samples and lung organoids and processed as bulk as previously described (Steens et al., 2021; Budeus et al., 2024). In brief, RNA concentration and quality were measured using the Qubit fluorometer (Invitrogen, Waltham, MA) and the Agilent Bioanalyzer (Agilent, Santa Clara, CA) with the DNA HS assay. Library preparation was performed using Lexogen QuantSeq 3′ mRNA-Seq Library Prep Kit FWD, and the libraries were sequenced on a NextSeq500 (Illumina, San Diego, CA). Sequences were trimmed using TrimGalore with standard settings and aligned with hisat2 to hg38 with standard settings. Statistical analysis was performed with R using the R packages (Budeus et al., 2024; Sentek et al., 2024; Budeus et al., 2023). Quantitative real-time RT-PCR (qRT-PCR) following cDNA synthesis using QuantiTect Reverse Transcription (Qiagen, Hilden, Germany) was performed according to the manufacturer’s instructions and using specific deoxy-oligonucleotide primers (Klein et al., 2013). For single-cell RNA sequencing analysis (scRNAseq), single-cell suspensions were generated by re-suspending LuOrgs in TrypLE (Thermo Fisher Scientific; #12604013) containing 100 µ/mL DNase I, followed by a 15-min incubation at 37 °C. Digestion was stopped by adding PBS containing 2%–5% fetal calf serum (FCS), 2 mM EDTA, and DNAse I. The cellular solution was passed through a 70-μm cell strainer into a fresh conical tube and labeled using the BD® Hu Single-Cell Multiplexing Kit (BD, Bioscience; #633781), according to the manufacturer’s instructions. Afterward, combined samples were loaded on lanes on a BD Rhapsody HT Xpress System as previously described (Budeus et al., 2024). BD Rhapsody library preparation was performed according to the manufacturer’s instructions, and the libraries were sequenced on a NextSeq 2000 using a P3 100-cycle kit. BDs cwl-runner was used to align and pre-analyze the data. All further analyses were conducted using R packages (Budeus et al., 2024; Buchholzki et al., 2025).

### Expression in HLCA

Single-cell data from healthy individuals in “The integrated Human Lung Cell Atlas (HLCA) v1.0” (Sikkema et al., 2023) were downloaded as h5ad file via CELLxGENE (https://cellxgene.cziscience.com/collections/6f6d381a-7701-4781-935c-db10d30de293), and normalized counts were converted into an Seurat single cell object in R. (In brief, SeuratDisks Convert function was used to convert into a h5seurat file, which could then be loaded into R. The original meta.data had to be processed separately and added afterward). We used the annotation structure presented in the atlas (levels 1–3).

### Statistical analysis

If not otherwise indicated (n = biological replicates), data were obtained from at least three independent experiments. Data analyses were performed using the Wilcoxon signed-rank test with R (R Core Team). Statistical significance was set at the level of p ≤ 0.05.

## Results

### HOX gene expression pattern in normal lung tissue

**FIGURE 1:**
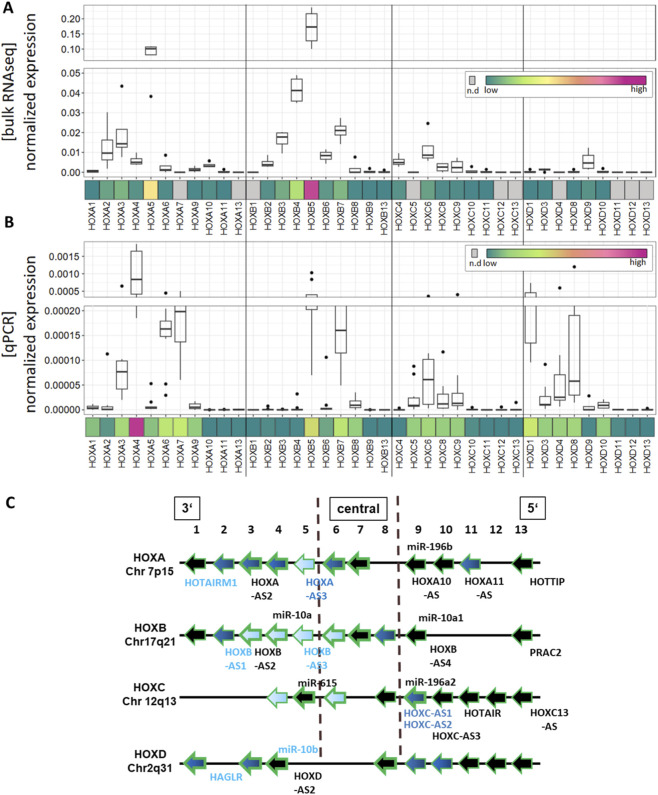
The lung HOX code. **(A)** Normalized expression of the 39 human HOX genes in whole normal lung tissue homogenates as obtained from RNA sequencing (bulk RNAseq) datasets. N = 4 biological replicates. **(B)** Expression levels of all HOX genes as quantified by real-time RT-PCR (qPCR). Respective transcript levels are shown as relative expression to beta-actin (set as 1). N = 4–8 biological replicates per gene. Individual symbols depict different biological replicates. **(C)** Scheme of the HOX gene expression pattern (the potential “lung HOX code”). Highly expressed genes as determined from bulk RNAseq are colored in light blue. Darker blue tones indicate lower expression. Black arrowheads indicate no expression. The green outline indicates validation by qPCR; the level of expression is shown by the thickness of the green line. The position of non-coding RNAs that are interspersed within the coding HOX genes is listed (miR, microRNA; AS, antisense RNA). Relevant expression levels (bulk RNA seq only) are marked accordingly in shades of blue.

The expression pattern of the known human HOX genes were first investigated in global gene expression profiles following RNA sequencing of whole lung tissue extracts generated from normal lung tissues (Figure 1). Transcript levels of the HOX candidates revealed expressions particularly of HOXA and HOXB cluster genes, namely, high mRNA expression levels of HOXA5 and HOXB5 and lower levels of HOXA2, HOXA3, HOXB3, HOXB4, HOXB6, and HOXB7, together with HOXC4, HOXC8, HOXC9, and HOXD9 from the HOXC and HOXD clusters (Figure 1A). HOXA4, HOXA6, and HOXA10, together with HOXB2 and HOXB8 mRNAs, were only detected at minimal levels. The lncRNA HOTAIRM1 located between the HOXA1 and HOXA2 genes, HOXB-AS1 located between the HOXB2 and HOXB3 genes, and HOXB-AS3 located between the HOXB5 and HOXB6 genes were accordingly found to be expressed, together with HAGLR located between the HOXD1 and HOXD3 genes (Supplementary Figure S1A). HOXA-AS3 (located between the HOXB5 and HOXB6 genes), as well as HOXC-AS1 and HOXC-AS2 (located between the HOXC9 and HOXC10 genes), and the regulatory molecule called microRNA-10b (miR-10b; located between the HOXD3 and HOXD4 genes) expressions were also detected, although at lower levels. To confirm the obtained sequencing results and thus to specify a potential “overall” lung HOX code, we performed qRT-PCR analysis of all human HOX genes using the whole lung RNA isolates and including more biological replicates (Figure 1B). The most abundantly expressed HOX genes detected here were again HOXA and HOXB cluster genes, with HOXA4 and HOXB5 showing the highest expression levels, followed by HOXA3, HOXA6, HOXA7, HOXB7, and HOXD1. HOXA2, HOXB6, HOXC6, and HOXD8 were detected at lower levels. The HOX genes HOXB4, HOXB8, HOXC5, HOXC6, and HOXC9, together with HOXD3 and HOXD4, were only detected at marginal levels. Surprisingly, HOXA5 expression was hardly detectable using qRT-PCR in whole lung RNA lysates in contrast to HOXA7, which was not detected by RNA sequencing. To verify the expression of both HOXA genes, the corresponding proteins were successfully detected via Western blot analysis (Supplementary Figure S1B).

**FIGURE 2:**
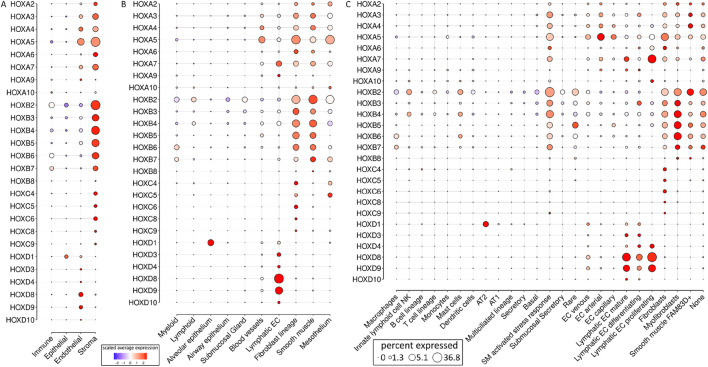
Gene expression of HOX genes in “The integrated Human Lung Cell Atlas (HLCA) v1.0.” Annotation of cell clusters was taken directly from Atlas data (Sikkema et al., 2023). Only healthy samples were used (HLCA core), and no cells were excluded. **(A)** Gene expression among annotation level 1; **(B)** gene expression among annotation level 2; **(C)** gene expression among annotation level 3. Size of the dots indicates the percentage of cells in which this gene was found, the color indicates the normalized value of the expression.

To assign the detected HOX expression patterns to specific lung cell types, we initially utilized publicly available RNA single-cell sequencing datasets. A Human Lung Cell Atlas (HLCA) was generated in a notable study based on numerous single-cell RNA datasets (Sikkema et al., 2023). From this, we used the integrated “HLCA core” dataset, which contains data on healthy lung tissue from 107 individuals (including raw and normalized counts, integrated embedding, cell-type annotations, and clinical and technical metadata) (Sikkema et al., 2023). Of the 39 HOX genes, five (HOXA13, HOXC11, HOXC12, HOXC13, HOXD12, and HOXD13) were not represented (Figure 2). An initial, broader classification [Level 1 (Sikkema et al., 2023)] into immune, epithelial, endothelial, and stromal compartments revealed that HOX expression is predominantly found in the stromal compartment (Figure 2A). The majority of the HOXA and HOXB cluster genes represented here are anterior and central HOX genes; the same applies—albeit at lower expression levels—to genes of the HOXC cluster. With the exception of HOXD1, which can be assigned to the epithelial compartment, HOXD gene expression was observed in the endothelium. Further specification of cell types [Levels 2–3 (Sikkema et al., 2023)] revealed that the expression of the HOXA genes HOXA3, HOXA4, HOXA5, HOXA6, and HOXA7, as well as the HOXB genes HOXB2, HOXB3, HOXB4, HOXB5, HOXB6, and HOXB7, was observed in fibroblast lineage cells (fibroblasts and myofibroblasts) and smooth muscle cells (Figures 2B,C). The expression of the HOXC genes HOXC4, HOXC5, HOXC6, HOXC8, and HOXC9 was also observed in fibroblasts, albeit to a lesser extent. Regarding HOXD gene expression, HOXD1 can be attributed to type 2 alveolar epithelial cells (AECII and AT2), whereas the expression of HOXD3, HOXD4, HOXD8, HOXD9, and HOXD10 was observed in endothelial cells—predominantly those of the lymphatic origin (Figures 2B,C).

To further confirm cell-type-dependent HOX expression patterns using qRT-PCR, we investigated HOX expression levels of the highest four genes per cluster (Figure 1B) in cultured lung epithelial cells, fibroblasts, and endothelial cells (Supplementary Figure S1C). Of the genes in the HOXA and HOXB clusters, which generally exhibited higher expression levels than the corresponding genes in the HOXC and HOXD clusters, only HOXA4, HOXA6, HOXB6, and HOXB7 could be detected in human bronchial epithelial cells (HBEC and BEAS-2B) and human small-airway epithelial cells (HSAEC), whereas HOXA3 and HOXA7 were expressed exclusively in HBECs. Similarly, low HOXC9 levels could be detected in bronchial epithelial cells. HOXC6 and HOXC8, together with HOXD1, although the latter is expressed only to a very limited extent, could indicate a HOX pattern more characteristic of the epithelium of the small airways. Overall, among the selected HOX genes, HOXA4, HOXA7, HOXC6, HOXC8, HOXC9, and HOXD8 exhibited higher expression levels in connective tissue cells [mesenchymal stem cells (MSCs) and fibroblasts (FIB)]. Furthermore, HOXA3 expression was most prominent in MSCs, and HOXD4 expression was highest in fibroblasts. Within endothelial cells, HOXB7 exhibited the highest expression levels, followed by HOXA4, HOXA6, HOXA7, HOXB5, HOXC5, and HOXD8.

In summary, based on the RNA expression analyses conducted so far, the HOXA and HOXB gene candidates appear to be the more relevant HOX genes in the lung overall. Regardless of the cell of origin, a characteristic HOX expression pattern—and thus a potential lung HOX code—can be specified (Figure 1C). HOXA3, HOXA4, HOXA6, and HOXA7, along with HOXB5 and HOXB7, were highly expressed in normal lung tissue, followed by the genes HOXC5, HOXC6, HOXC8, and HOXC9, as well as HOXD1 and HOXD8, which showed lower levels. Although HOXA5 showed only low mRNA expression levels, its relevance has been confirmed at the protein level. Regarding the cell-type specificity of the detected HOX expression, HOX genes of the HOXA and HOXB clusters were found predominantly in stromal cell types (fibroblasts and smooth muscle cells), with mesenchymal cells also representing the relevant cell types for HOXC expression. Candidates from the HOXD group appeared to be restricted primarily to mesenchymal cells and small-airway epithelial cells, which may suggest a more distal cellular identity. Infiltrating immune cells generally exhibit (very) low HOX expression levels.

### HOX protein localization in normal lung tissue

**FIGURE 3:**
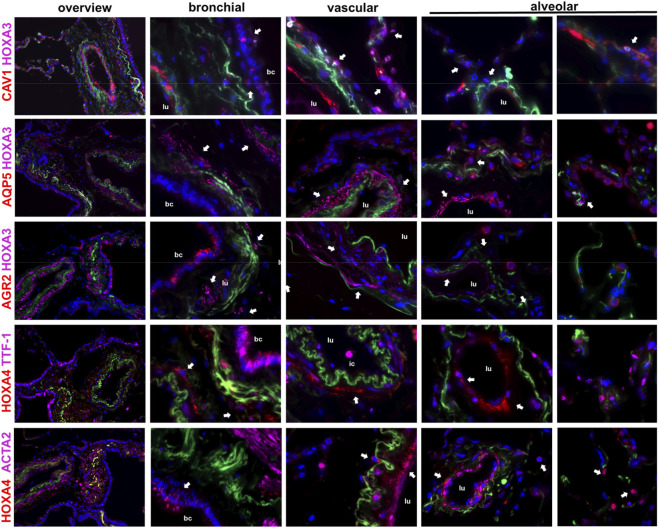
Immunofluorescent analysis of HOXA proteins (I). Immunofluorescent staining of normal lung sections was performed using antibodies against the HOXA proteins HOXA3 and HOXA4, together with antibodies against the indicated marker antigens. CAV1 (caveolin 1), which is also known to be expressed by vascular cells, and AQP5 (aquaporin-5) were used as markers for AECI, and TTF-1 (thyroid transcription factor-1, also known as NK2 homeobox 1, NKX2-1) was used as a marker for AECII. AGR2 (anterior gradient protein 2 homolog) was used as a marker for bronchial epithelial (particularly goblet) cells, and ACTA2 (smooth muscle aortic alpha-actin) was used as a classical smooth muscle cell/myofibroblast marker. The autofluorescence properties of the extracellular matrix protein fibers (shown in green) were included to better visualize the morphology of the lung structures shown. Nuclei were visualized using Hoechst 33342 nucleic acid stain (blue). Representative lung photographs of bronchial, vascular, and alveolar structures are shown (magnifications). Arrows highlight HOX-immunoreactive structures. bc, bronchial epithelium; lu, lumen; ic, immune cell. Single-channel images of the merged pictures are depicted in the Supplementary Figures.

**FIGURE 4:**
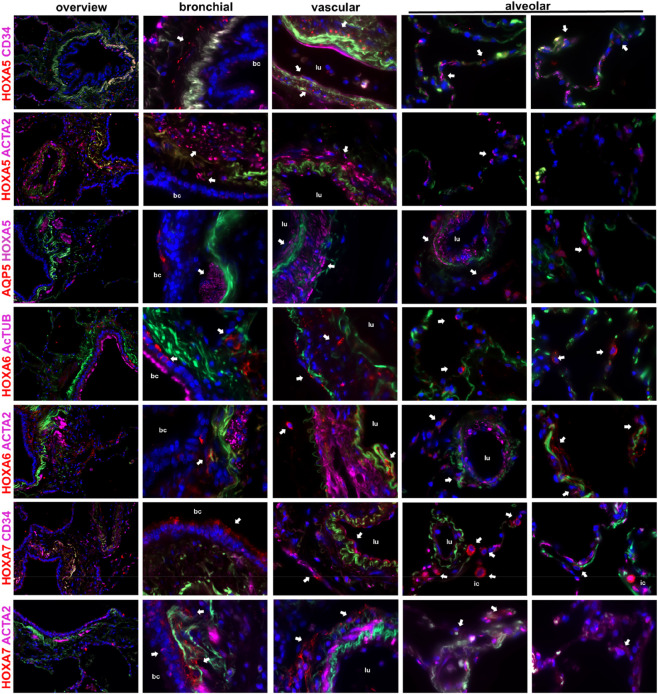
Immunofluorescent analysis of HOXA proteins (II). Immunofluorescent staining of normal lung sections was performed using antibodies against the HOXA proteins HOXA5, HOXA6, and HOXA7, together with antibodies against the indicated antigens. AcTUBA (acetylated alpha-tubulin) was used as a ciliated cell marker, and the glycosylated transmembrane protein CD34 as a marker for endothelial cells. Nuclei were visualized in blue. Representative lung photographs of bronchial, vascular, and alveolar structures are shown (magnifications). Arrows highlight HOXA-immunoreactive structures. bc, bronchial epithelium; lu, lumen; ic, immune cell. Single-channel images of the merged pictures are depicted in the Supplementary Figures.

**FIGURE 5:**
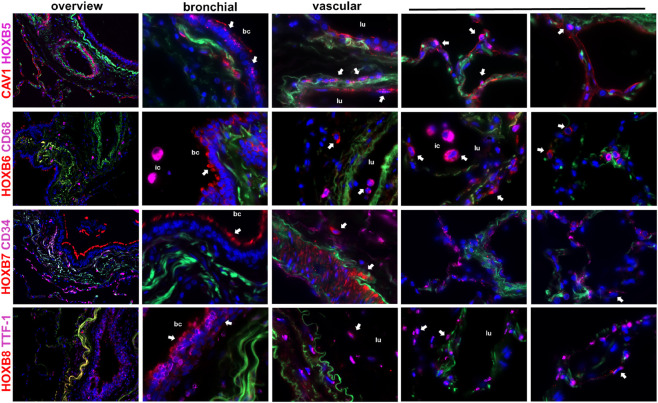
Immunofluorescent analysis of HOXB proteins. Immunofluorescent staining of normal lung sections was performed using antibodies against the indicated HOXB proteins, together with antibodies against the indicated antigens. The glycoprotein CD68 was used as a marker for monocyte lineage cells and tissue macrophages. Nuclei were visualized using Hoechst 33342 nucleic acid stain (blue). Representative lung photographs of the different lung structures are shown (magnifications). Arrows highlight HOXB-immunoreactive structures. bc, bronchial epithelium; lu, lumen; ic, immune cell. Single-channel images of the merged pictures are depicted in the Supplementary Figures.

**FIGURE 6:**
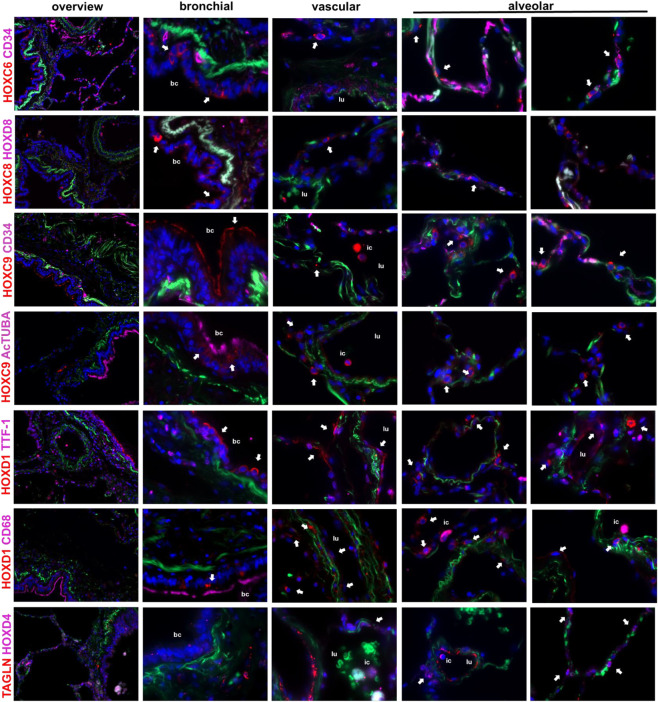
Immunofluorescent analysis of HOXC and HOXD proteins. Immunofluorescent staining of normal lung sections was performed using antibodies against the indicated HOXC or HOXD proteins, together with antibodies against the indicated antigens. Tagln (transgelin) was used as a marker for smooth muscle cells. Nuclei were visualized in blue. Representative lung photographs of bronchial, vascular, and alveolar structures are shown (magnifications). Arrows highlight HOXC/D-immunoreactive structures. bc, bronchial epithelium; lu, lumen; ic, immune cell. Single-channel images of the merged pictures are depicted in the Supplementary Figures.

Next, using available antibodies and immunohistochemical analysis, we examined the expression patterns of HOX proteins—corresponding to the previously identified lung HOX genes—in samples of normal human lung (Figures 3–6). HOXA3 protein expression could be detected in nuclei of single bronchiolar epithelial cells. Pronounced cytoplasmic immunoreactivity was observed in subbronchial and vascular smooth muscle cells, as well as in endothelial cells of larger blood vessels, although the immunoreactivity was rather weak in the latter. HOXA3 expression was further detectable in certain (CAV1-positive) cells within the alveolar region, which could indicate expression in alveolar fibroblasts (Figure 3; Supplementary Figures S2–S5). Unfortunately, mesenchymal markers could not be co-localized (not shown). Nuclear HOXA4 expression was prominent in single bronchial cell clusters and single alveolar epithelial cells (Figure 4; Supplementary Figures S2, S6, S7). Strong cytoplasmic HOXA4 immunoreactivity was observed in cells beneath bronchi, bronchioles, and larger blood vessels, indicating localization in SMCs and connective tissue cells. A similar connective tissue/SMC-associated immunoreactivity was also observed for HOXA5, together with a clear, albeit more cytoplasmic, localization in endothelial cells (again) of larger blood vessels (Figure 4; Supplementary Figures S2, S8–S10). Single cells located within the alveolar region also showed faint HOXA5 immunoreactivity. HOXA6 protein expression was observed beneath bronchial regions, in areas associated with blood vessels, and in specific cells of the bronchial epithelium (Figure 4; Supplementary Figures S2, S11, S12). The lack of co-localization with the SMC marker ACTA2 indicated clear expression in the connective tissue. Furthermore, prominent immunoreactivity in larger alveolar cells with a typical AECII morphology could be detected. However, the overall expression was cytoplasmic and not nuclear. HOXA7 protein expressions seemed to be more ubiquitous. Bronchial epithelial cells altogether were stained within the ciliated region; HOXA7 expression showed a more nuclear localization in simple columnar epithelial cells of terminal bronchioles or cuboidal epithelial cells of respiratory bronchioles and thus in more distally localized epithelial cells (Figure 4; Supplementary Figures S2, S13, S14). Immunoreactivity could further be observed in endothelial cells and connective tissue cells, where only a weak co-localization with ACTA2-positive cells could be observed. Although both cytoplasmic and nuclear localization could be observed, the cytoplasmic localization was more prominent. Within the alveolar region, only very few HOXA7-immunoreactive cells could be visualized, which also lacked ACTA2-immunoreactivity. Infiltrating immune cells further showed HOXA7-immunoreactivity.

Concerning the HOXB candidates, HOXB5, HOXB6, HOXB7, and HOXB8 were investigated (Figure 5; Supplementary Figure S15). HOXB5 expression was clearly localized to vascular mural and endothelial cells, with a more cytoplasmic localization (Figure 5; Supplementary Figures S15, S16). Within bronchial epithelial cells, cytoplasmic HOXB5 expression was prominent in the luminal region. In addition, single HOXB5-positive cells localized within the adventitia and within the alveolar interstitium could be detected that could resemble more undifferentiated mesenchymal cells (Steens et al., 2021; Klein, 2021). HOXB6 expression was detected in bronchial epithelial cells in luminal regions, where it seemed to be cilia-associated (Figure 5; Supplementary Figures S15, S17). Single-cell clusters within the laminar propria also showed non-nuclear HOXB6 staining. These cells could originate from sections of seromucous glands. Furthermore, HOXB6 was found to be localized in tissue-associated cells that were not properly integrated. Together with co-localization of the immune cell marker CD68, this expression accounted for the presence of infiltrating immune cells and alveolar macrophages. HOXB7 expression was prominent in larger bronchial structures where the antibody appeared to be trapped extracellularly to the cilia (Figure 5; Supplementary Figures S15, S18). A concrete (cytoplasmatic) cellular staining was—similar to HOXB5—detectable in stabilizing vascular SMCs, but not in sub-bronchial SMCs. Single HOXB7-positive cells were localized within the adventitia. Within the alveolar compartment, HOXB7 was hardly detectable. HOXB8 expression was detectable within the luminal region of ciliated bronchial epithelial cells (Figure 5; Supplementary Figures S15, S19). Weak immunoreactivity was observed in vascular SMCs and some single alveolar cells.

From the HOXC cluster, HOXC6, HOXC8, and HOXC9 were investigated as there was no functional antibody—at least in our hands—for HOXC5 (Figure 6; Supplementary Figure S20). HOXC6 showed cytoplasmic localization in single bronchial epithelial cells, in single cells found in the connective tissue close to larger blood vessels, and in single cells at the blood–air interface (Figure 6; Supplementary Figures S20, S21). Together with its known expression in mesenchymal stem cells (Steens et al., 2021; Klein, 2021; Sentek and Klein, 2021), these patterns suggest generally a more connective tissue association. Similarly, HOXC8 showed a partial immunoreactivity concerning bronchial epithelial cells and a weak cytoplasmic expression in connective tissue regions and some alveolar cells (Figure 6; Supplementary Figures S20, S22). HOXC9 showed rather unspecific binding at the cilia surface and weak immunoreactivity within the cytoplasm of bronchial cells (Figure 6; Supplementary Figures S20, S23, S24). A specific (cytoplasmic) localization of HOXC9 was observed within larger alveolar cells. Regarding the larger cell size, these cells are likely to be AECII cells, together with single (infiltrating) immune cells showing strong HOXC9 immunoreactivity.

Concerning HOXD proteins, HOXD1 expression could be detected in the luminal regions of bronchial epithelial cells, in endothelial cells, and in single cells associated with the alveolar interstitium. Together with the observed expression in some loosely associated cells, the latter one could be indicative for alveolar macrophages (Figure 6; Supplementary Figures S20, S25, S26). Unfortunately, we were not able to detect HOXD3. HOXD4 expression was somehow restricted to single—again larger—alveolar epithelial cells and some infiltrating immune cells (Figure 6; Supplementary Figures S20, S27). In addition, weak endothelial immunoractivity was observed. HOXD8 expression was hardly detectable by immunofluorescence in normal human lung tissue (Figure 6; Supplementary Figures S20, S22). In a few—rather squamous—alveolar epithelial cells, partial nuclear HOXD8 expression could be detected.

In summary, based on the observed immunoreactivity and consistent with the HOX RNA expression patterns described above, the HOXA and HOXB candidates are predominantly localized in stromal cells. Although HOXA3 and HOXA5 were primarily localized in sub-bronchial and vascular SMCs, HOXA4, HOXB5, and HOXB7 were predominantly found in vascular SMCs, whereas expression in sub-bronchial regions was more associated with connective tissue. HOXA6 was not observed in ACTA2-positive cells but was detected in sub-bronchial and adventitial connective tissue; a similar pattern was observed for HOXA7. HOXB8 was detected in vascular SMCs only, with very weak immunoreactivity. HOXA7, HOXB5, and HOXB7, along with HOXD1, were found in the endothelial cells of predominantly larger vessels. Bronchial epithelial cells showed immunoreactivity for almost all the HOX proteins examined, although in some cases, this was limited to isolated cells. In the alveolar compartment, only HOXA6 (presumably in connective tissue cells), as well as HOXB6, HOXC6, and HOXC8, could be localized.

### HOX gene expression pattern in iPSC-derived lung organoids

**FIGURE 7:**
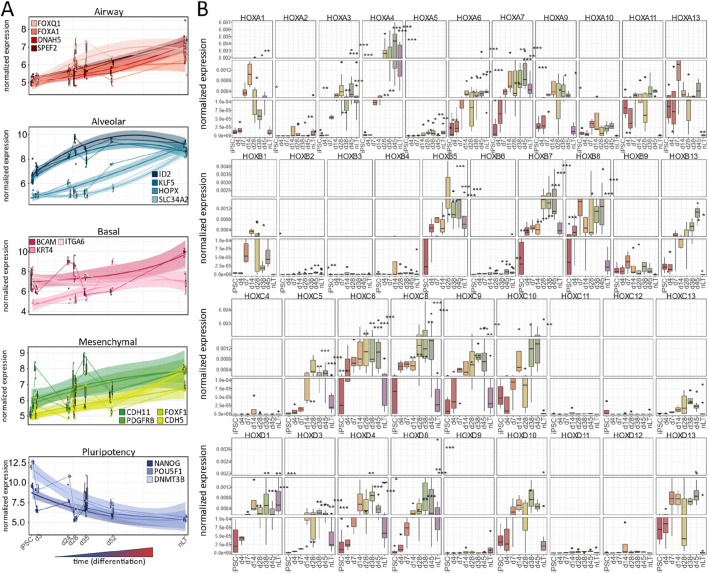
HOX expression levels in developing lung organoids. **(A)** Normalized expression of selected genes (bulk RNA sequencing) categorized as airway, alveolar, basal, mesenchymal, and pluripotency genes in iPSCs (day 0) and differentiating lung organoids compared to normal lung tissue (nLT). Trend is calculated by local polynomial regression fitting (Loess). N = 4 biological replicates N = 4 biological replicates per time point. **(B)** Quantitative real-time RT-PCR of HOX expression levels in iPSCs (day 0, d0), generated lung organoids at d4, d7, d14, d28, d38 and d45 compared to nLT. Respective transcript levels are shown as relative expression to beta-actin (set as 1). Individual symbols depict different biological replicates (n = 4–8 per gene and timepoint). Wilcoxon–Mann–Whitney test * = 0.05, ** = 0.01, and *** = 0.001 (compared to iPSC, d0).

Finally, we aimed to examine how the HOX expression observed in adult lung tissue develops during lung development (Figure 7). For this purpose, we used our previously published datasets generated from developing lung organoids (LuOrg) derived from human iPSCs (Budeus et al., 2024; Budeus et al., 2025). RNAseq analysis of whole organoid RNA extracts revealed upregulation of various lung cell type-specific genes during lung organoid differentiation, such as FOXQ1, FOXA1, DNAH5, and SPEF2 for bronchial epithelial cells, along with BCAM TP63, KRT4, and ITGA6 for basal cells including BASC (Figure 7A; Supplementary Figure S28). The formation of alveolar cell types was indicated by the upregulation of ID2, KLF5, HOPX, and SLC34A2. Classical mesodermal genes such as CDH11, FOXF1, and PDGFRB were also upregulated during the differentiation period, as is the endothelial marker CDH5, while downregulation of pluripotency genes was observed. The mRNA levels of all 39 HOX genes were then quantified by qRT-PCR, starting with the iPSCs (day 0). The course of HOX expression during differentiation was recorded on days 4, 7, 14, 28, 38, and 45 compared to adult lung tissue (nLT) (Figure 7B). Increased expression levels of the HOXA genes HOXA3, HOXA4, HOXA6, and HOXA7 were detected during differentiation, reaching levels comparable to those in normal lung tissue (Figure 7B). HOXA5 showed a similar pattern, although its expression levels were significantly lower. HOXA2 was expressed at only very low—presumably negligible—levels. Interestingly, HOXA1 levels increased during differentiation (days 7–14), but decreased again at later time points. Elevated levels of HOXA9 and HOXA10 were also observed during differentiation, but these levels were inconsistent with the low expression levels found in normal lung tissue. HOXA11 and HOXA13 showed higher, albeit overall low, expression levels compared to nLT, but these levels were already present in the initial cells. Among the HOXB genes, the levels of HOXB5, HOXB7, and HOXB8 showed an increase in expression over time, consistent with the nLT levels. Similar to HOXA1, HOXB1 also appeared to play a role in the development of lung structures. Here, an increase in expression was observed, which then decreased again over time. HOXB13, on the other hand, was induced during iPSC-LuOrg differentiation but was not present in nLT. HOXB2, HOXB3, HOXB4, and HOXB9 expression were difficult to detect, as were the expressions of the HOXC and HOXD genes HOXC4, HOXC11, HOXC12, HOXD9, HOXD11, and HOXD12. HOXC5, HOXC6, HOXC8, and HOXC9 expression levels increased during LuOrg differentiation and were comparable to levels in normal lung tissue. HOXC10 levels, however, showed an increase during differentiation that was not reflected in the nLT. Similarly, HOXD1, HOXD3, HOXD4, and HOXD8 showed an increase that closely matches the values in normal lung tissue. An increase in HOXD10 and HOXD13 levels can also be observed; however, this contradicts the observed nLT values.

**FIGURE 8:**
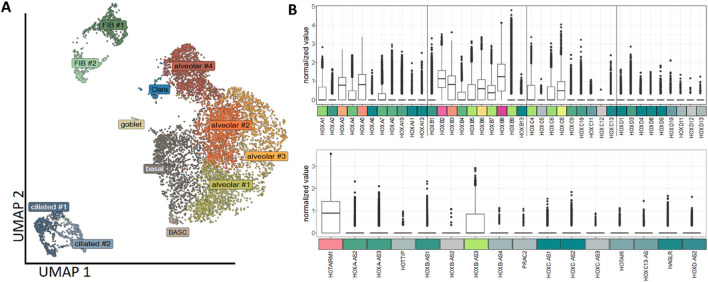
Single-cell RNA sequencing analysis of human lung organoids. **(A)** Uniform Manifold Approximation and Projection (UMAP) for dimension reduction, colored by identified clusters of all single cells generated from human lung organoids. N = 3 biological replicates. Cell-type labels for each cluster are based on the expression of canonical cell-type markers. For details, we refer to the *Results* section and the respective manuscript (Budeus et al., 2024). **(B)** Normalized HOX gene and related lncRNA/miR expression levels in whole (cluster-independent) lung organoids obtained from own previously published single-cell RNA sequencing datasets (Budeus et al., 2024).

**FIGURE 9:**
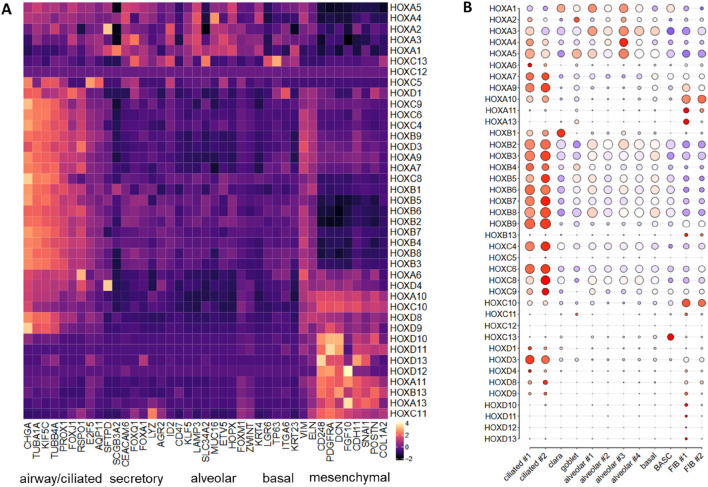
Cell-type-specific HOX gene expression levels in lung organoids. **(A)** Row-normalized heatmap of the 39 human HOX genes as expressed in cells characterized by the indicated marker genes for airway, alveolar, basal, and mesenchymal cells. **(B)** Dot plot of the HOX genes as expressed by the cells of the different clusters. Size of the dots indicates the percentage of cells in which this gene was found, and the color indicates the normalized value of the expression.

Overall, the HOX expression levels developing in the lung organoid model confirm the potential lung HOX code found in adult lung tissue. However, it should be noted that certain lung cell types are not present within lung organoids, such as resident (and infiltrating) immune cells or a generally high number of endothelial cells, which are only found sporadically in this model (Budeus et al., 2024; Budeus et al., 2025). Furthermore, the observed differences in HOX expression patterns and levels could also be due to the relatively young age of the organoids compared to adult lung tissue as aging can alter or reduce HOX expression levels. Nevertheless, to further specify the findings on the cell type-specific HOX expression patterns resulting from the immunohistochemical analyses, we examined the RNA expression levels of the HOX genes in datasets of iPSC-derived lung organoids after a single-cell analysis. In sufficiently matured lung organoids, 12 cell clusters can be distinguished, including classical airway, alveolar, and fibroblast cell subsets (Figure 8A). Basal cells expressing ITGA6, TP63, and LGR6, as well as bronchioalveolar stem cells (BASCs) characterized by the additional co-expression of bronchiolar (CCNO) and alveolar (SLC34A2 and AQP3) epithelial genes, were identified close to four alveolar cell clusters. Cluster alveolar #1 consists of AECI/II lineage-prone cells (AECI/II-differentiating BASC cells) expressing HOPX, ETV5, SLC34A2, KRT23, and MUC15, in addition to more advanced differentiated alveolar cells designated based on the additional expression of GABRP, LAMP3, and CAV1 (alveolar #2). AECII (alveolar #3) cells were found to express increased levels of LAMP3 and GABRP levels, together with high-molecular-weight glycoproteins (MUC16 and MUC20), while HOPX levels were reduced. The alveolar #4 cluster was designated as the AECI cluster based on the expression of FOXM1, RTKN2, and ZWINT and its direct vicinity to alveolar fibroblasts (FIB #1) expressing FOXM1, together with FGF10, SNAI1, FOXF1, and other classical mesodermal genes. Adventitial fibroblasts (FIB #2) showed increased expression levels of more reactive extra cellular matrix-remodeling-related genes, particularly DCN, ELN, POSTN, and CD248. Four (larger) airway cell clusters were further identified: two TUBA1A- and TUBB2B-expressing ciliated (#1 and #2) cell clusters, with cluster ciliated #1 (RFX4, FOXJ1, and RSPH1) comprising luminal epithelial cells undergoing ciliogenesis and with cluster ciliated #2 comprising more mature ciliated cells (TUBB4A and PROX1). Secretory goblet cells and Clara (club) cells were identified by the expression of LYZ, ARG2, MUC3A, TSPAN8, and CEACAM6, along with SCGB3A2, SFTPD, and FOXA3. In the entire dataset—initially independent of cell type—HOX expression of HOXA1, HOXA3, HOXA4, HOXA5, and HOXA7, as well as the HOXB genes HOXB2 to HOXB9, HOXC4, HOXC6, and HOXC8, were detected at higher levels (Figure 8B). Some HOX genes, such as HOXA9, HOXA10, HOXB1, and HOXB9, along with some late HOXC and HOXD genes, showed high expression in individual cells (individual black dots), but not on average across all cells, i.e., they only affect a few (<10%) cells. Expression of the lncRNAs HOTAIRM1 and HOXB-AS3 was further detected. Consequently, we then investigated which LuOrg cells these expression levels originated from (Figure 9; Supplementary Figure S29). Examining the expression levels of HOX genes in cells that classically express marker genes for (bronchial) airway (ciliated, secretory Clara, and goblet cells), alveolar, and finally mesenchymal cells revealed that (Figure 7A) ciliated cells highly expressed many HOXB genes, as well as the previously identified HOXC candidates. In secretory airway, bronchial, and basal cells, the anterior HOXA genes were more prominent, while in mesenchymal cells, i.e., fibroblasts, the posterior genes were predominant. This was also reflected when examining HOX expression in the previously identified cell clusters (Figure 7B). In ciliated cells, a potential HOX code appears to encompass numerous HOX genes, particularly the posterior HOXA gene, almost all HOXB genes, central HOXC, and anterior HOXD1 genes. HOXA1, along with HOXB1, could be a marker for the numerically less numerous Clara cells. No convincing HOX code is evident for goblet cells. HOXA2 and HOXA5 could play a role here. The anterior HOXA genes HOXA1, HOXA3, and HOXA5 were prominently expressed in the alveolar clusters, especially in AECII cells (alveolar cluster #3), together with HOXA4, whereas expression in AECI/II lineage-prone cells was not yet as pronounced, which could indicate that this AECII HOXA code is still evolving. In basal cells, no HOX gene is clearly predominant. The situation was similar in BASCs, with the exception of HOXC13, which is found to be more highly expressed in BASC. Both fibroblast clusters showed increased expression of posterior genes, particularly HOXA10, HOXA11, and HOXC10, in addition to HOXA11 and HOXA13 in the alveolar (FIB #2) cluster.

## Discussion

The tissue dependence of HOX activity, which manifests itself in phenotypic expressions, is based on the regulation of cell-dependent target genes and genetic networks (Rezsohazy et al., 2015; Cain and Gebelein, 2021). Identification of characteristic organ- and cell-type-specific expression patterns of HOX genes could not only reveal the topological origin of cell types but also lead to new therapeutic approaches if changes in HOX gene expression during disease could be correlated with (pathological) alterations in corresponding cells, the manipulation of which would then be of enormous importance for limiting tissue degeneration and/or promoting regeneration. One prerequisite for this is deciphering the HOX code of individual tissues. Here, we have focused on the potential HOX code of healthy lungs. Based on mRNA expression levels, combined with studies on the localization of candidate HOX proteins, it was found that HOXA and HOXB genes belonging to the early and central groups are expressed at higher levels overall in lung tissue—predominantly in stromal cell types. Bronchial epithelial cells showed more complex immunoreactivity for almost all the HOX proteins examined, although in some cases, this was limited to single cells here. The HOXB proteins HOXB5 and HOXB7 could be localized to the vascular compartment, together with HOXD1, which was predominately localized to endothelial cells. Overall, HOX expression levels were lower in the alveolar compartment.

Analyzing HOX gene expression patterns and levels in normal human lung tissue already revealed that HOX genes predominantly from the anterior 3′ end of the HOXA and HOXB clusters were expressed in adult lungs (Golpon et al., 2001). In addition to high HOXA5 expression levels that was found in alveolar septal and epithelial cells, HOXB2 and HOXB6 were the most abundant HOX genes (Golpon et al., 2001). We did not investigate HOXB2, but HOXB6 expression was only detected sporadically, predominantly in alveolar or infiltrating immune cells. For HOXA5, and similarly for HOXA4, we observed connective tissue- and SMC-associated immunoreactivity, together with a clear, albeit more cytoplasmic, localization in bronchial epithelial cells. Accordingly, HOXA5 gene expression, and even more general expression of HOX5 paralogs, was specifically found within lung mesenchymal cells, particularly in stromal fibroblasts (Li et al., 2021; Jeannotte et al., 2016; Boucherat et al., 2013). Mesodermal HOXA5 is now recognized as a known important factor in normal lung alveoli that suppresses inflammation as HOXA5 deficiency triggered elastic fiber abnormalities accompanied by the recruitment of inflammatory cells to the alveoli, together with goblet cell anomalies, ultimately resulting in goblet cell hyperplasia (Mandeville et al., 2006; Pai and Sukumar, 2020). HOXA4 and HOXA5 are also known as vascular-associated HOXA genes being important in the processes of vascular remodeling (Zhou et al., 2024). In addition, HOXA4, HOXA5, and HOXC9 might also be important during Clara-like cell differentiation (Yoshimi et al., 2005). In contrast, in adult lungs, HOXA4 and HOXA5 were suggested as epithelial-related HOX genes as both HOXA genes presented a high level of protein expression in nucleus of epithelial cells in normal alveoli and bronchial structures, while significant downregulation was observed in the lung cancer epithelium (Gao et al., 2022). Although the cellular origin was not investigated, HOXA2, HOXA3, HOXA4, HOXA5, and HOXA6, together with HOXB3, HOXB4, and HOXB5, were confirmed by qRT-PCR analysis to be expressed at higher levels in normal human lung tissue, with HOXB5 showing the highest levels, when HOX expression levels in normal lung tissue, primary lung tumors, and derived lung cancer cell lines were compared (Plowright et al., 2009). HOXA6 and HOXA7 protein expressions seemed to be more ubiquitous, being expressed in sub-bronchial and adventitial connective tissue and partially bronchial epithelial cells. We detected mostly negligible expression levels for all posterior genes, which is in line with previous investigations. Among the posterior HOXA and HOXB genes, particularly HOXA9, HOXA10, and HOXB9 were found to be upregulated in several lung cancer cell lines and, consistently, in lung tumors, while normal lungs were shown to lack HOXA9 and HOXA10 expressions (Calvo et al., 2000). Of note, only low expression levels of HOXA5 were estimated (Calvo et al., 2000).

Generally, posterior HOX gene expression is known to be relevant in mesodermal cells, particularly fibroblasts. Gene expression in combination with corresponding methylation analysis already revealed a potential HOX code for fibroblasts, which included the posterior HOX genes HOXA11 and HOXA13, as well as HOXD1, HOXD10, and HOXD13 (Budeus et al., 2023). Furthermore, HOXB9, HOX13, HOXC4, and HOXC12 could serve as additional, albeit less important, HOX code candidates in fibroblasts. Similarly, the posterior HOX genes HOXA/D9, HOXA/D10, HOXA/D11, and HOXA13 were found to be highly expressed in adult fibroblasts (Pfeiferová et al., 2025). Using single-cell RNA-sequencing of human lymphangioleiomyomatosis lung tissue, which is characterized by proliferation of abnormal smooth muscle-like cells, revealed a unique panel of signature genes selectively expressed including the posterior HOX genes HOXA10, HOXA11, and HOXD11 that were not or rarely detectable in normal lung tissue (Guo et al., 2020; Olatoke et al., 2022). In contrast, mesenchymal cells within the adventitia, namely, vascular wall-resident mesenchymal stem cells, which might be considered fibroblast progenitors, mainly expressed central HOX genes, together with HOX genes neighboring closely the central cluster region: HOXB7, HOXC6, and HOXC8, together with HOXB5 (Steens et al., 2021; Klein et al., 2013; Steens and Klein, 2022; Steens et al., 2019). A prominent role of HOXA3, in addition, was revealed concerning the therapeutic potential of these adventitial cells (Budeus et al., 2023). Distinct expression of HOXA9, HOXA10, HOXB7, HOXC6, HOXC8, HOXC10, and HOXD8 can frequently be observed in multipotent stromal cells derived from different human tissues, which along with their central role in defining positional identity, might impact on controlling organ-specific structures (of the stroma) and thus cell types through a tissue-specific combination of mechanisms (Steens and Klein, 2022; Picchi et al., 2013; Smith et al., 2019; Kulebyakina and Makarevich, 2020).

HOX expression analysis in adult normal lung tissue further revealed that human lungs showed relatively high expression of anterior HOXB cluster genes, particularly HOXB2, HOXB3, HOXB4, HOXB5, and HOXB6, together with HOXA3, whereas relatively low or no expression of posterior HOX cluster genes (paralogs 9–13) was detected (Abe et al., 2006). HOXB5–HOXB7 expression was confirmed in our present study, and the HOXB5 protein, similar to HOXB7, was clearly localized to vascular mural and endothelial cells. In addition, single HOXB5-positive cells localized in the sub-bronchial adventitia and within the alveolar interstitium were detected, which may represent more undifferentiated mesenchymal cells (Steens et al., 2021; Klein, 2021). HOXB8 expression was weakly detectable within ciliated cells of the bronchial epithelium but clearly detectable in AECII cells. For HOXC and HOXD cluster genes, generally quite low expression levels in lungs were observed (Plowright et al., 2009). In addition to the central HOXC candidates, suggesting more mesodermal cells of origin, levels of HOXD1, HOXD3, HOXD4, and HOXD8 could be estimated. These four HOXD candidates were already found to be restricted to endothelial cells, bearing a more microvascular phenotype, while macrovascular artery endothelial cells lack respective expressions (Toshner et al., 2014). For endothelial cells, a hierarchy of cellular identities unrelated to organ-specific identity was suggested as endothelial cells did not cluster with cells from similar segmental regions but instead clustered based on the type of blood vessels (Toshner et al., 2014). Moreover, persisting HOX expression patterns in post-natal tissues were particularly prominent in (highly) vascularized tissues such as the lungs, suggesting that HOX genes play a critical role in maintaining vascular homeostasis in a region-specific manner (Visconti and Awgulewitsch, 2015). In particular, a HOXD gene signature seems to reflect the differing origins of the endothelial cells. According to the expression of HOXD8 and HOXD9 in the angiogenic endothelium of the developing human fetus, a distinct endothelial expression of HOXD8 and HOXD9 within the developing lung was estimated, which coincided with the known peak of lung angiogenesis during the pseudoglandular and canalicular stages (Toshner et al., 2014).

Finally, a very elegant study generated a Human Lung Cell Atlas based on numerous single-cell RNA datasets (Sikkema et al., 2023). This created the possibility of analyzing the cell-type-specific expression of specific genes of interest when the corresponding complex analyses cannot be performed in one’s own research facilities. From this, we used the integrated “HLCA core” dataset, which contains data on healthy lung tissue to assign the detected HOX expression patterns to specific lung cell types. Here, it was clearly evident that the HOXA and HOXB candidate genes originate predominantly from stromal cells. Overall, it should be noted that the level of HOX gene expression in most of the single cells was very low to absent; for example, 75% of the clusters of annotation level 3 have a cell percentage expression of HOX genes below 1.2%. This could introduce a degree of uncertainty regarding these cell-type-specific HOX expression patterns. Further differences likely stem from the distinct single-cell RNA sequencing methodologies employed. Although the HLCA core single cell datasets are based on 10x Genomics technology (Sikkema et al., 2023), we used the BD Rhapsody HT Xpress System for the single-cell analysis of the lung organoids. For this reason, we opted against integration or label transfer between the two datasets.

Conclusively, HOXA3–HOXA7, along with HOXB5 and HOXB7, were highly expressed in normal lung tissue, followed by the genes HOXC6, HOXC8, and HOXC9, which showed overall lower expression levels, along with HOXD1–HOXD8. Some HOX candidates (e.g., HOXC5, HOXD3, and HOXD8) could not convincingly be localized at protein level using immunohistochemistry and immunofluorescence, which is probably due to a lack of reactivity and thus to imperfection of the antibodies. However, it is striking that most of the other HOX proteins were either not expressed in the nucleus or were only partially localized there. The nuclear signal were generally rather weak in contrast to the cytoplasmic localization; possibly, only small amounts of protein are required for transcriptional HOX activity. Overall, however, the described expressions were more cytoplasmic, which is rather surprising. The intracellular distribution of HOX proteins are known to display dynamic changes according to the developmental stages and/or differentiation steps in humans (Bridoux et al., 2021). Relocating HOX proteins to the cytoplasm, a supposed mechanism to regulate HOX activity, may result in either subsequent cytoplasmic degradation or sustained cytoplasmic stability, thereby enabling a readily mobilizable supply of HOX proteins to re-enter the nucleus (Bridoux et al., 2021). HOXB4, for example, was detected to be cytoplasmic throughout fetal epidermal development but essentially nuclear in the upper layers of adult skin (Kömüves et al., 2002). The nuclear exit of HOX proteins, in turn, relies on the interaction with exporting proteins; for example, interaction with KPC2 in the nucleus stimulates its (CRM1-dependent) nuclear exit (Bridoux et al., 2021; Bridoux et al., 2015). In the cytoplasm, de-ubiquitination of HOX proteins could contribute to their stabilization, and subsequent ubiquitination might promote HOX nuclear re-entry and transcriptional activity (‘ready-to-use supply’) (Bridoux et al., 2021; Bridoux et al., 2015). How individual HOX proteins remain stable and thus presumably active for longer periods in certain cell types, thereby influencing their fate and contributing to organ-specific (cellular) phenotypes, remains incompletely uderstood and requires further characterization, particularly of lung cell type-specific HOX protein activities and their respective cellular localization. HOX proteins are known to have functions beyond their roles as DNA-binding transcription factors. For example, HOXA13 and HOXD13 interact with SMAD proteins to modify SMAD transcriptional activation. The homeo-domain of HOX proteins was shown to bind to CREB-binding proteins, thereby blocking acetyltransferase activity (Williams et al., 2005; Shen et al., 2001), demonstrating that HOX proteins modulate the transcriptional activity through interactions with other proteins in a non-DNA-binding manner. Likewise, cytoplasmic binding of HOXA9 by SMAD4 and thus nuclear entry restrain of a HOX protein was identified as a protective mechanism to prevent HOXA9-induced transformation of normal hematopoietic stem and progenitor cells and subsequent excessive expansion (Quéré et al., 2011). Outside the nucleus, HOX proteins have also been implicated in other non-transcriptional functions, namely, in controlling mRNA splicing, translation, or modulating cell signaling (Rezsohazy, 2014; Draime et al., 2018). Expression of alternative HOX mRNAs at different differentiation stages might result in distinct protein isoforms, e.g., lacking the homeodomain and, in turn, influencing localization (Bridoux et al., 2021; Fernandez and Gudas, 2009; LaRosa and Gudas, 1988). An incomplete or truncated HOX protein could presumably be preferentially localized in the cytoplasm, where it engages in various protein–protein interactions and exhibits specific molecular activities, whereas the spliced transcript encoding the complete HOX protein might later be (re)-induced when needed.

Thus, aspects of the molecular biology of HOX proteins (including activity regulation at the post-translational level) remain insufficiently understood. This may be due, in part, to the fact that HOX proteins are primarily studied in the context of embryonic development. Non-coding RNAs (ncRNAs) and micro RNAs (miRs) might add another layer to the colinear expression of Hox genes (Parambil et al., 2025). ncRNAs are important post-transcriptional regulators that modulate HOX gene expression by interacting with respective mRNA, thereby promoting degradation and/or inhibiting protein translation (Jasim et al., 2024). Likewise, HOX genes serve as transcriptional regulators of specific miR and lncRNAs. This complex interplay is crucial not only for the precise regulation of gene expression during development but also for establishing tissue-specific patterns. The disruption of HOX–lncRNA/miR interactions, however, can significantly impact in normal tissue homeostasis and also disease pathogenesis (Novikova and Kulakova, 2021), particularly relevant to various cancers as aberrant expression of HOX genes, interspersed lncRNA, and miR have become a defining feature in numerous cancer types (Rawat et al., 2020; Shao et al., 2021). For example, miR-10 b is encoded in HOXD, together with the two antisense lncRNAs, HOXD-AS2 and HAGLR/HOXD-AS1 (Gonçalves et al., 2020). miR-10b was shown to impact on neighboring HOXD3 and HOXD4 expressions, and upregulation of HOXD-AS2 lncRNA was shown to drive HOXD3/D4/miR-10b expression (Deforzh et al., 2022). Accordingly, miR-10b, which was found to be expressed in lung tissue, could account for the reported HOXD expressions. Likewise, HOXA-AS3 expression was shown to increase HOXA6 mRNA stability by forming an RNA duplex (Zhang et al., 2018), which fits to the reported HOXA6 and HOXA-AS3 expressions.

The dependence on animal models, in combination with the reliance on early stages of embryogenesis, might have led to an under-appreciation of the extent of involvement of HOX genes in adult lung cell identity. The identification of certain factors and associated signaling pathways that bring about the correct spatial and temporal regulation of HOX gene expression in the lungs can then ultimately lead to controlling lung development via molecular analysis and manipulation of the components. However, research on HOX genes in general presents particular challenges. The high similarity of HOX proteins complicates the production of specific antibodies. Antibodies are not commercially available for all HOX proteins, and those that are vary in their efficacy for different applications. Furthermore, HOX genes may exhibit only low or highly restricted expression in a limited number of cells, both during development and in adult tissue, which complicates localization at the protein level using immunofluorescence. Additionally, current single-cell technologies may not yet provide sufficient resolution to accurately detect HOX gene expression. Here, we provide further insights into the HOX expressions of normal lung tissue and thereby propose a preliminary lung HOX code. Nevertheless, further, specifically functional, studies are needed to align the cell-type-specific expression and localization of HOX proteins with cellular phenotypes. The comparatively high immunoreactivity of AECII cells with respect to the different HOX code candidates could already be indicative that these cuboidal defenders of the alveolus with secretory characteristics might serve as facultative progenitors for epithelial regeneration.

## Data Availability

The datasets presented in this study can be found in online repositories. The names of the repository/repositories and accession number(s) can be found in the article/Supplementary Material.
