# Supplementary material for: Toward a cell-type-specific lung HOX code
Source: Front Cell Dev Biol. 2026 Jul 14;14:1839422. doi: 10.3389/fcell.2026.1839422 (PMC13407532; doi:10.3389/fcell.2026.1839422)
Supplement: Supplementary file 1 [file DataSheet1.pdf]

Supplemental Marterial

Towards a cell-type specific lung HOX code

Bettina Budeus[1], Diana Klein[1,*]

[1]Institute for Cell Biology (Cancer Research), Medical Faculty, University of Duisburg-Essen, Essen, Germany

* Correspondence:

Prof. Dr. rer. nat. Diana Klein; Diana.Klein@uk-essen.de

(https://orcid.org/0000-0002-1770-443X)

Availability of data and material

The RNA-seq data (bulk and single cell) have been deposited at Gene Expression Omnibus (GEO) and are publicly available as of the date of publication (accession number: GSE275539). Any additional information required to reanalyze the data reported in this paper is available from the corresponding author upon request.

Supplemental Figures

Supplemental Figure S1

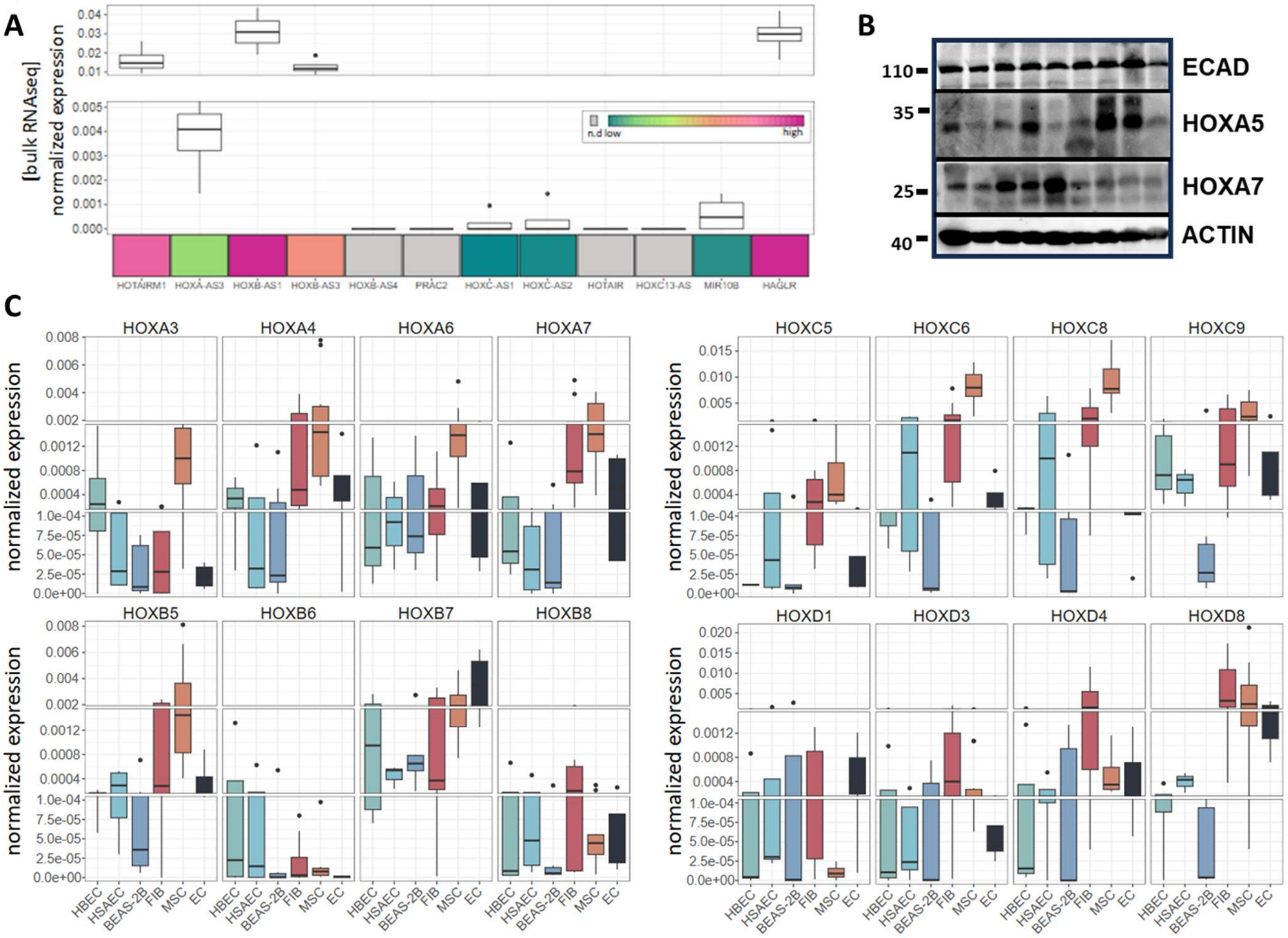

Lung-relevant HOX genes. (A) Normalized expression of the indicated lncRNA/ miR in whole normal lung tissue homogenates as obtained from RNA sequencing data sets. N = 4 biological replicates per condition. (B) Expression levels of the indicated proteins were analyzed in whole protein lysates of normal human lung tissue. Each lane depicts a different tissue homogenate (biological replicate). Beta-Actin was included as loading control. Molecular weight markers are indicated. Ful gels are depicted in Supplemental Figure S30. (C) Indicated transcript levels were quantified in total RNA extracts of cultured bronchial (HBEC, HBEC3-KT and BEAS-2B) and small airway (HSAEC, HSAEC1-KT) as well as (microvascular) endothelial cells (EC, HMEC-1) and fibroblasts (FIB, HS-5 and mesenchymal stem cells, MSC) using Real-Time RT-PCR and are shown as relative expression to beta-actin (set as 1).

Supplemental Figure S2

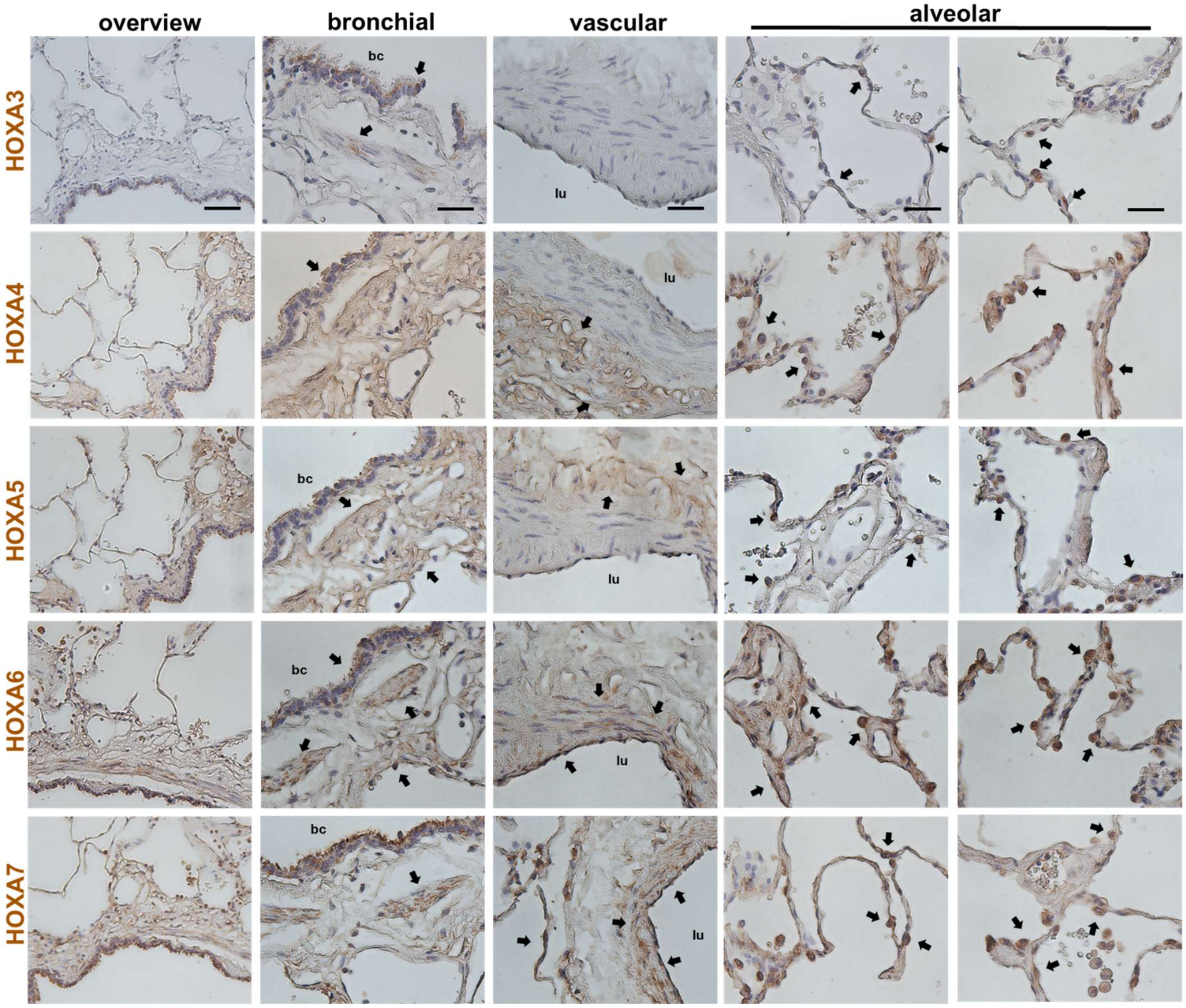

Immunohistochemical analysis of HOXA proteins. Immunohistochemical staining of normal lung tissue sections was performed using the indicated HOXA antibodies and DAB staining (brown). Nuclei were counterstained with hematoxylin (blue). Representative lung photographs of bronchial, vascular and alveolar structures are shown (magnifications). Arrows highlight immunoreactive structures. bc bronchial epithelium, lu lumen. Scale bar indicates 100 µm (left panel) and 10 µm (higher magnifications).

Supplemental Figure S3

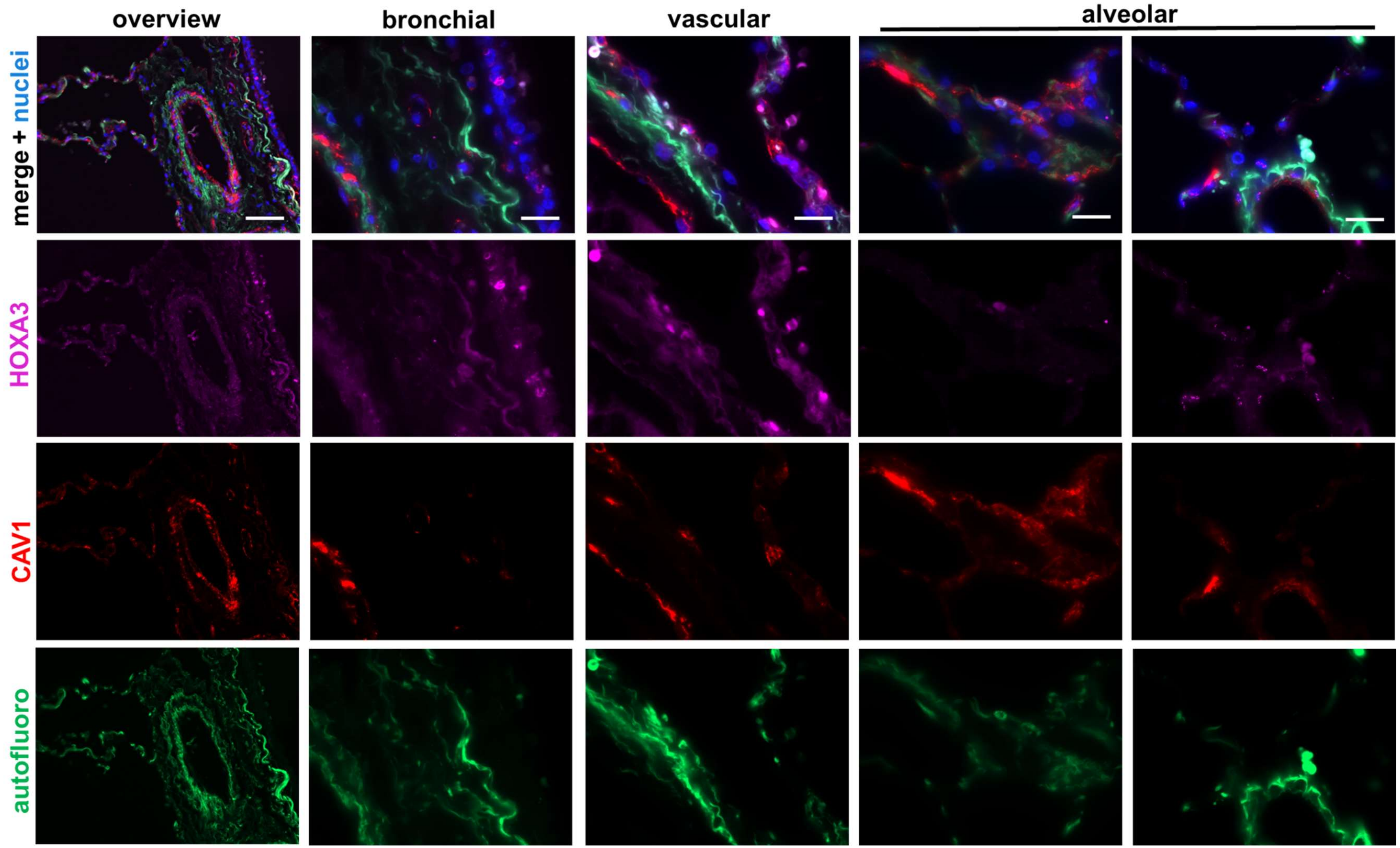

Immunofluorescent analysis of HOXA3 (I). Double-immunofluorescent staining of normal lung sections was performed using antibodies against HOXA3 (violet) and caveolin-1 (CAV1; red). Nuclei were visualized in blue. Single channel images of the merged pictures depicted in Figure 3 are shown. Scale bar indicate 100 µm (overview) and 10µm (higher magnifications).

Supplemental Figure S4

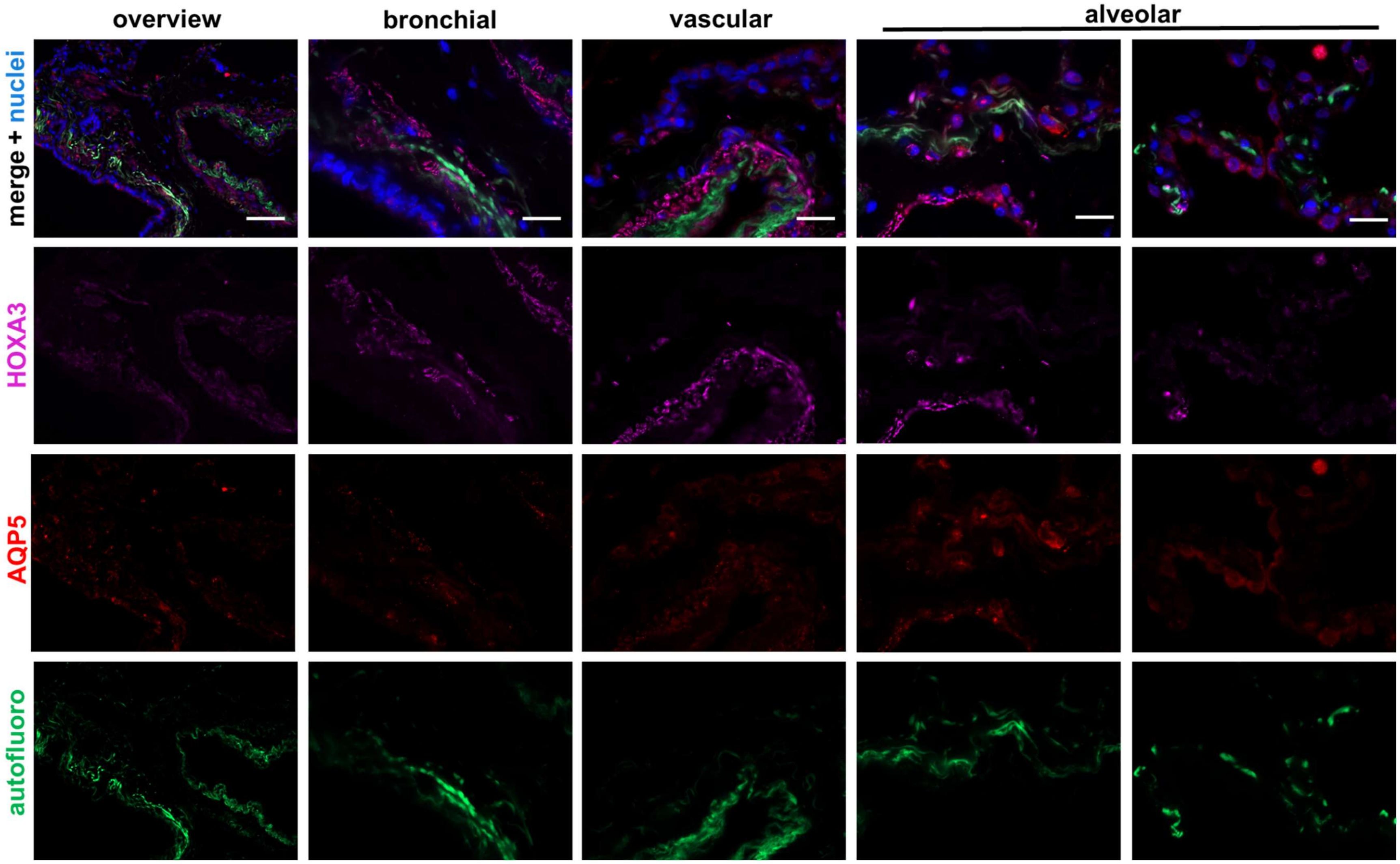

Immunofluorescent analysis of HOXA3 (II). Double-immunofluorescent staining of normal lung sections was performed using antibodies against HOXA3 (violet) and AQP5 (red). Nuclei were visualized in blue. Single channel images of the merged pictures depicted in Figure 3 are shown. Scale bar indicate 100 µm (overview) and 10µm (higher magnifications).

Supplemental Figure S5

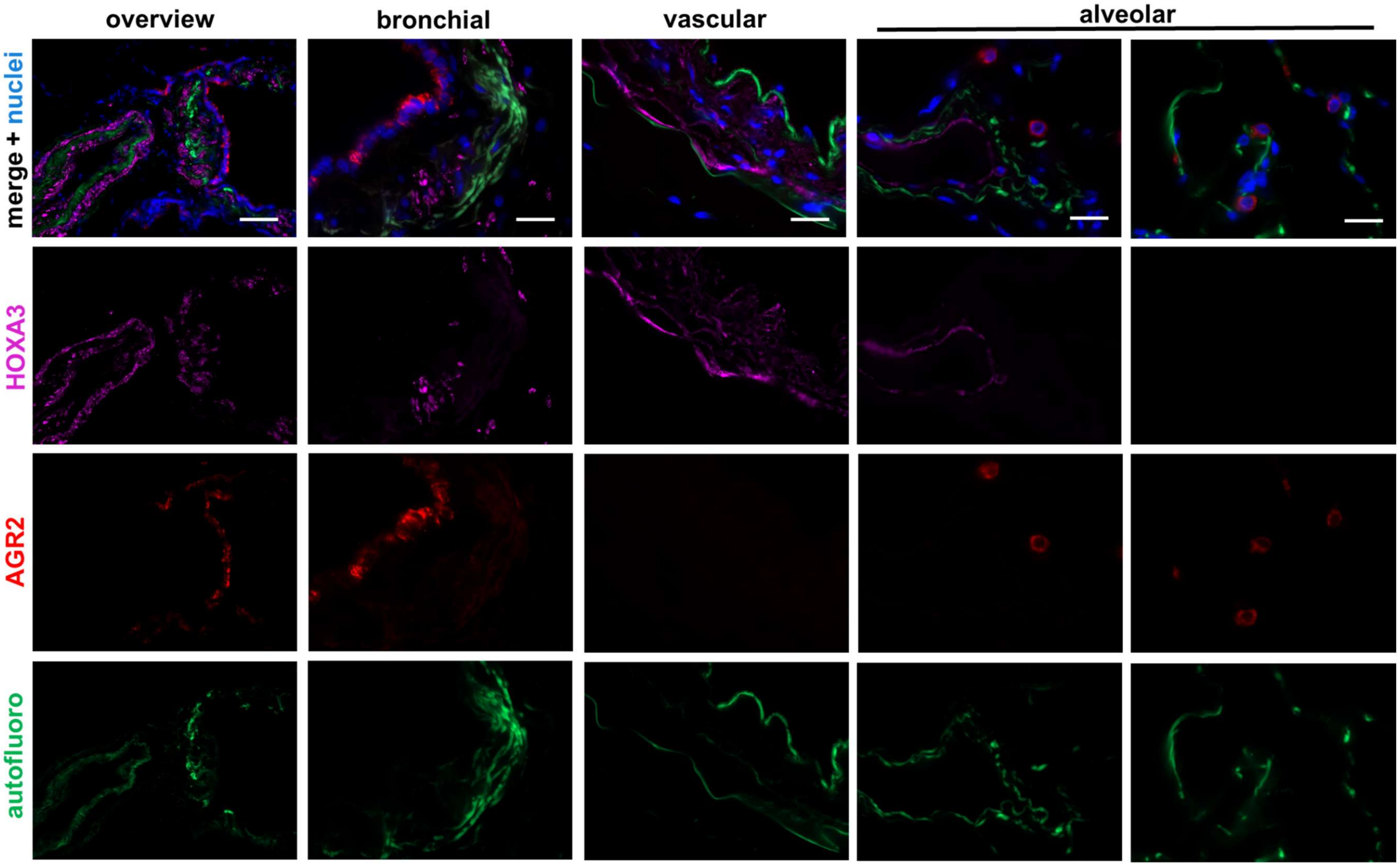

Immunofluorescent analysis of HOXA3 (III). Double-immunofluorescent staining of normal lung sections was performed using antibodies against HOXA3 (violet) and AGR2 (red). Nuclei were visualized in blue. Single channel images of the merged pictures depicted in Figure 3 are shown. Scale bar indicate 100 µm (overview) and 10µm (higher magnifications).

Supplemental Figure S6

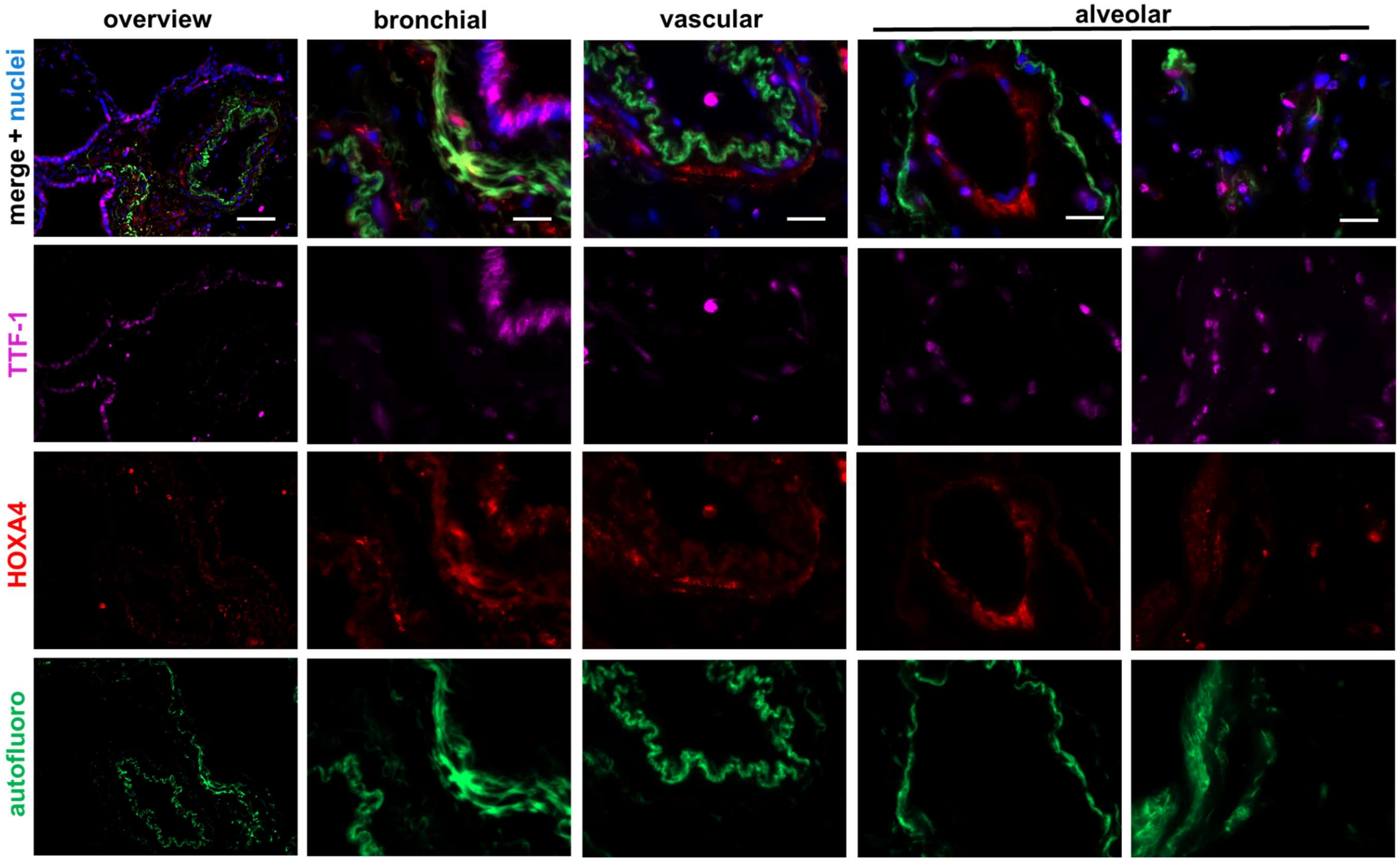

Immunofluorescent analysis of HOXA4 (I). Double-immunofluorescent staining of normal lung sections was performed using antibodies against HOXA4 (red) and TTF-1 (violet). Nuclei were visualized in blue. Single channel images of the merged pictures depicted in Figure 3 are shown. Scale bar indicate 100 µm (overview) and 10µm (higher magnifications).

Supplemental Figure S7

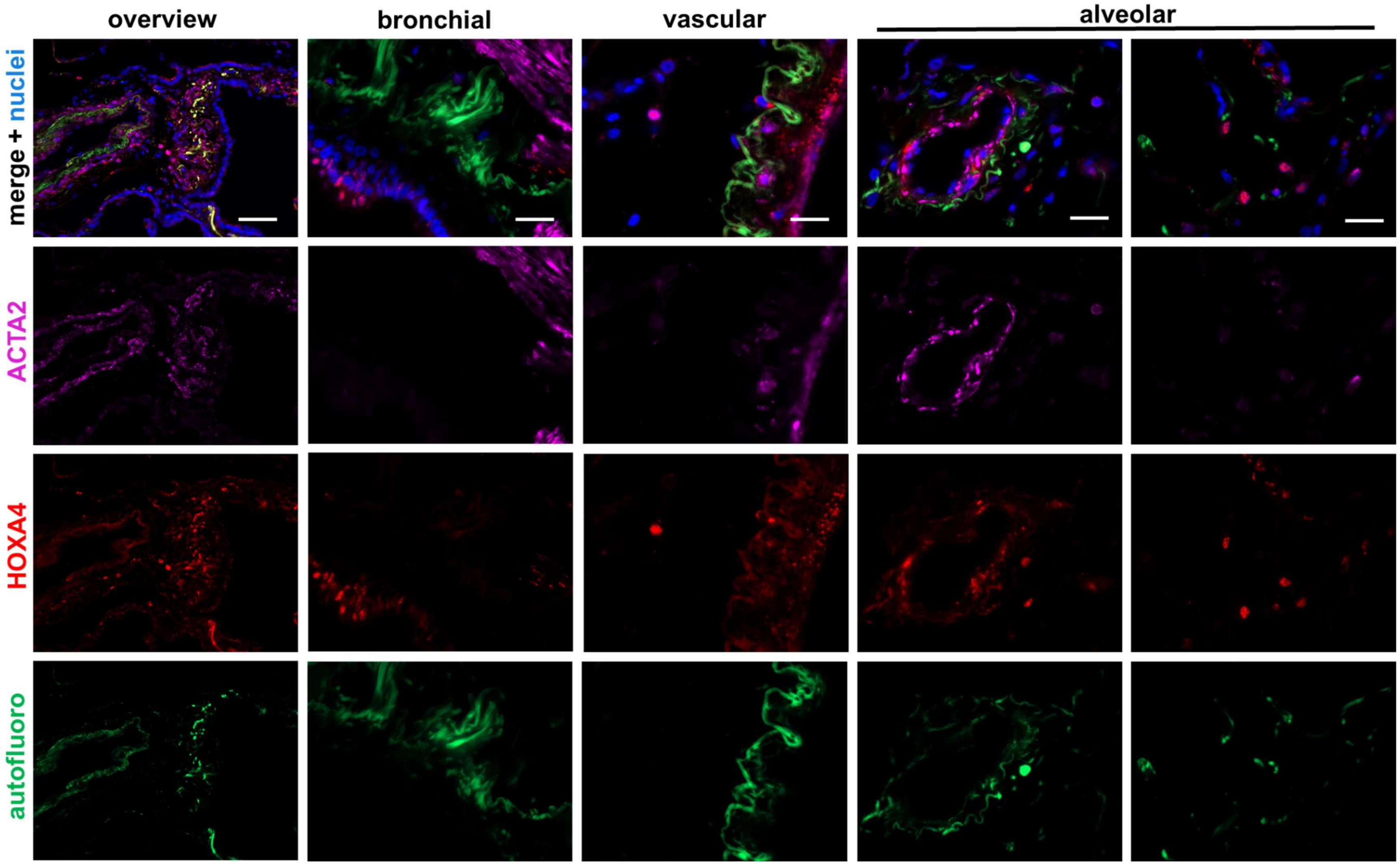

Immunofluorescent analysis of HOXA4 (II). Double-immunofluorescent staining of normal lung sections was performed using antibodies against HOXA4 (red) and ACTA2 (violet). Nuclei were visualized in blue. Single channel images of the merged pictures depicted in Figure 3 are shown. Scale bar indicate 100 µm (overview) and 10µm (higher magnifications).

Supplemental Figure S8

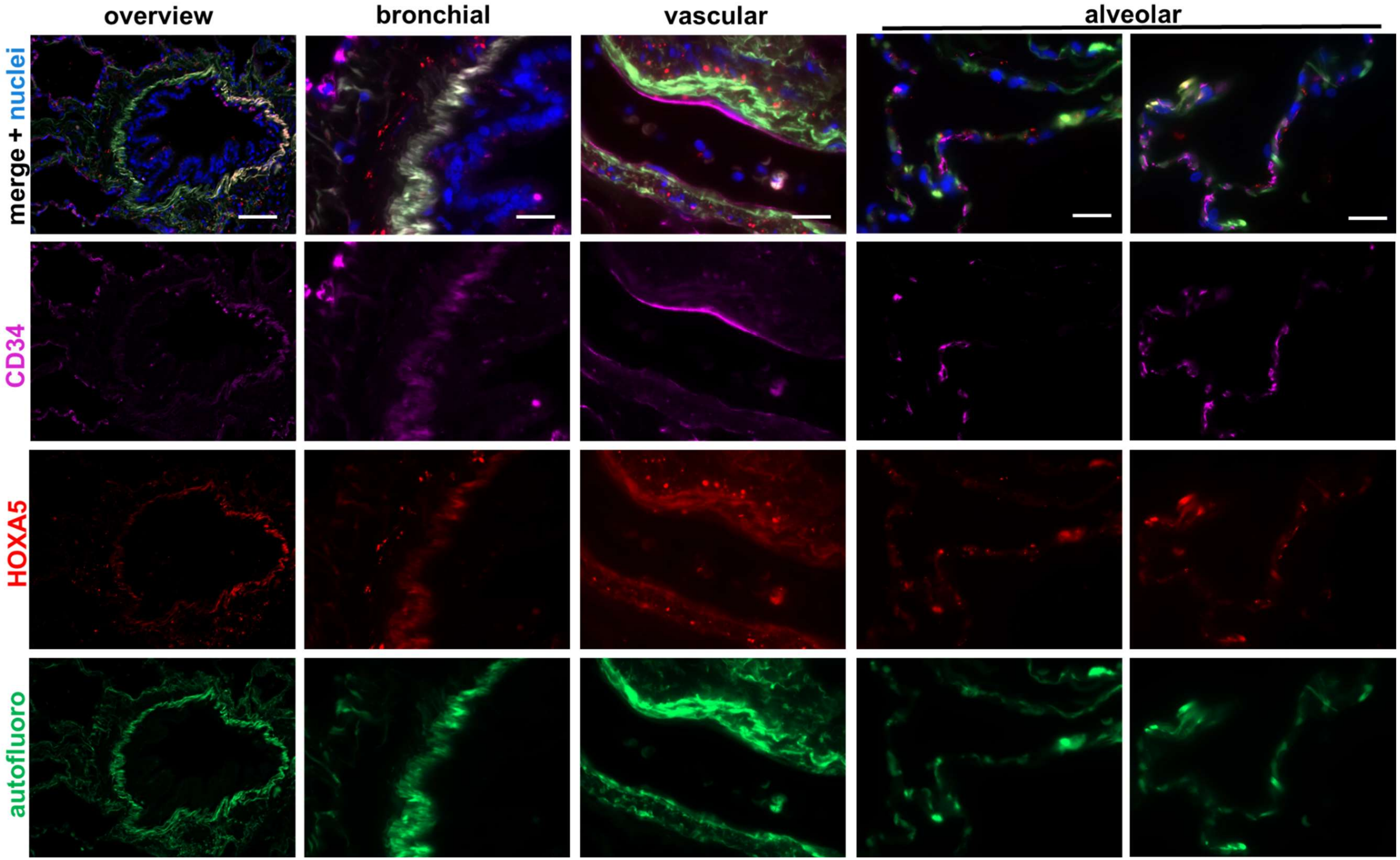

Immunofluorescent analysis of HOXA5 (I). Double-immunofluorescent staining of normal lung sections was performed using antibodies against HOXA5 (red) and CD34 (violet). Nuclei were visualized in blue. Single channel images of the merged pictures depicted in Figure 4 are shown. Scale bar indicate 100 µm (overview) and 10µm (higher magnifications).

Supplemental Figure S9

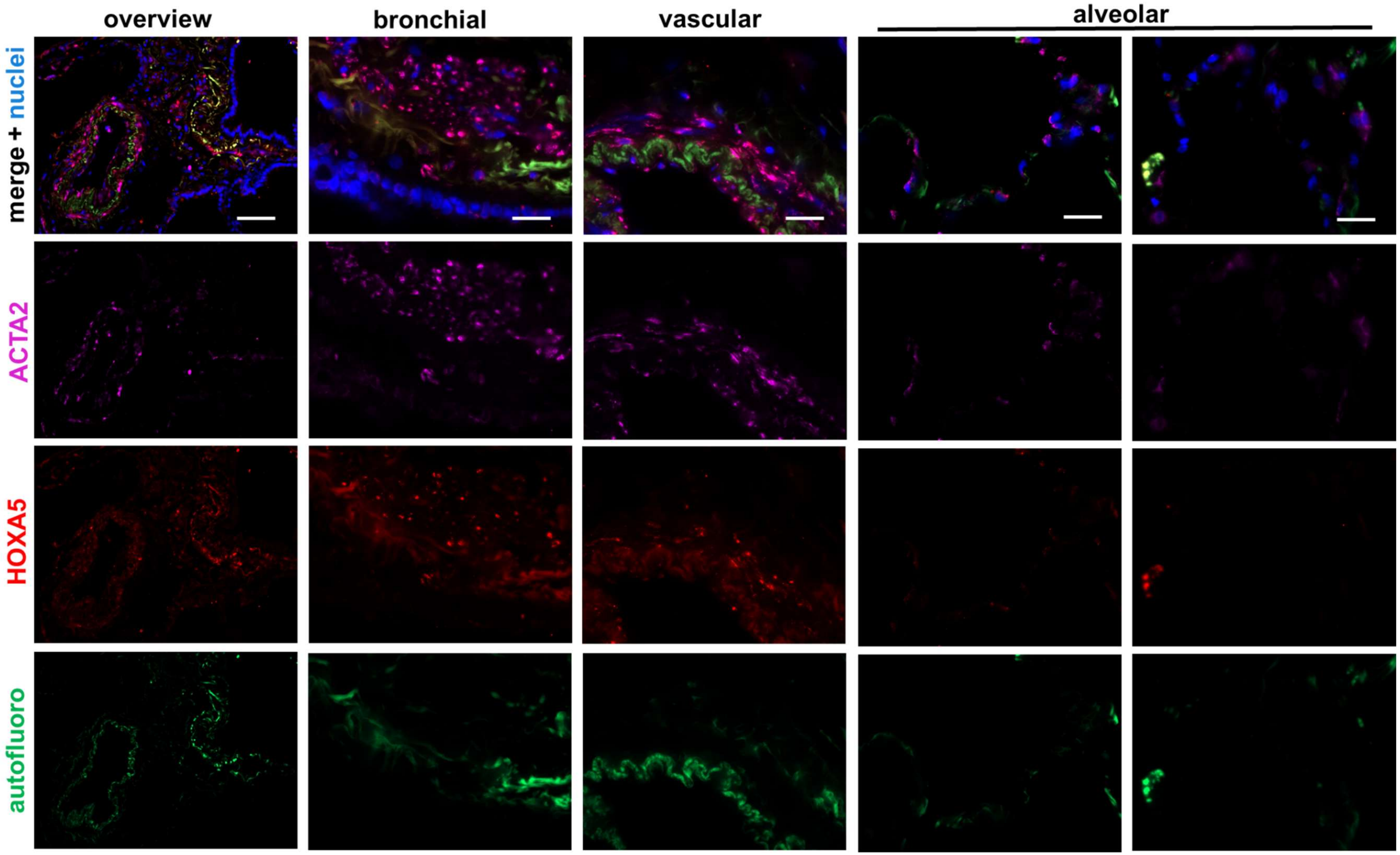

Immunofluorescent analysis of HOXA5 (II). Double-immunofluorescent staining of normal lung sections was performed using antibodies against HOXA5 (red) and ACTA2 (violet). Nuclei were visualized in blue. Single channel images of the merged pictures depicted in Figure 4 are shown. Scale bar indicate 100 µm (overview) and 10µm (higher magnifications).

Supplemental Figure S10

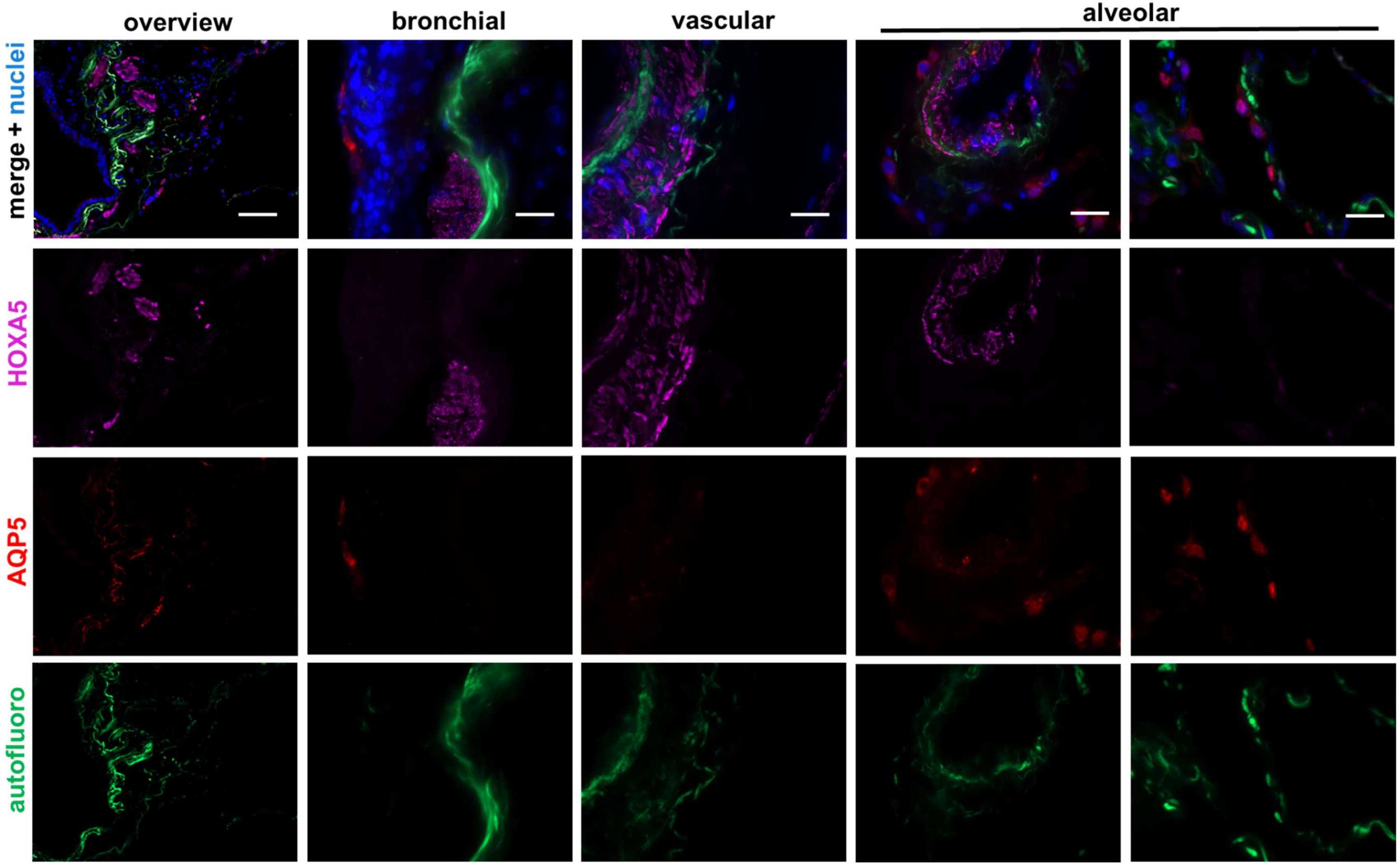

Immunofluorescent analysis of HOXA5 (III). Double-immunofluorescent staining of normal lung sections was performed using antibodies against AQP5 (red) and HOXA5 (violet). Nuclei were visualized in blue. Single channel images of the merged pictures depicted in Figure 4 are shown. Scale bar indicate 100 µm (overview) and 10µm (higher magnifications).

Supplemental Figure S11

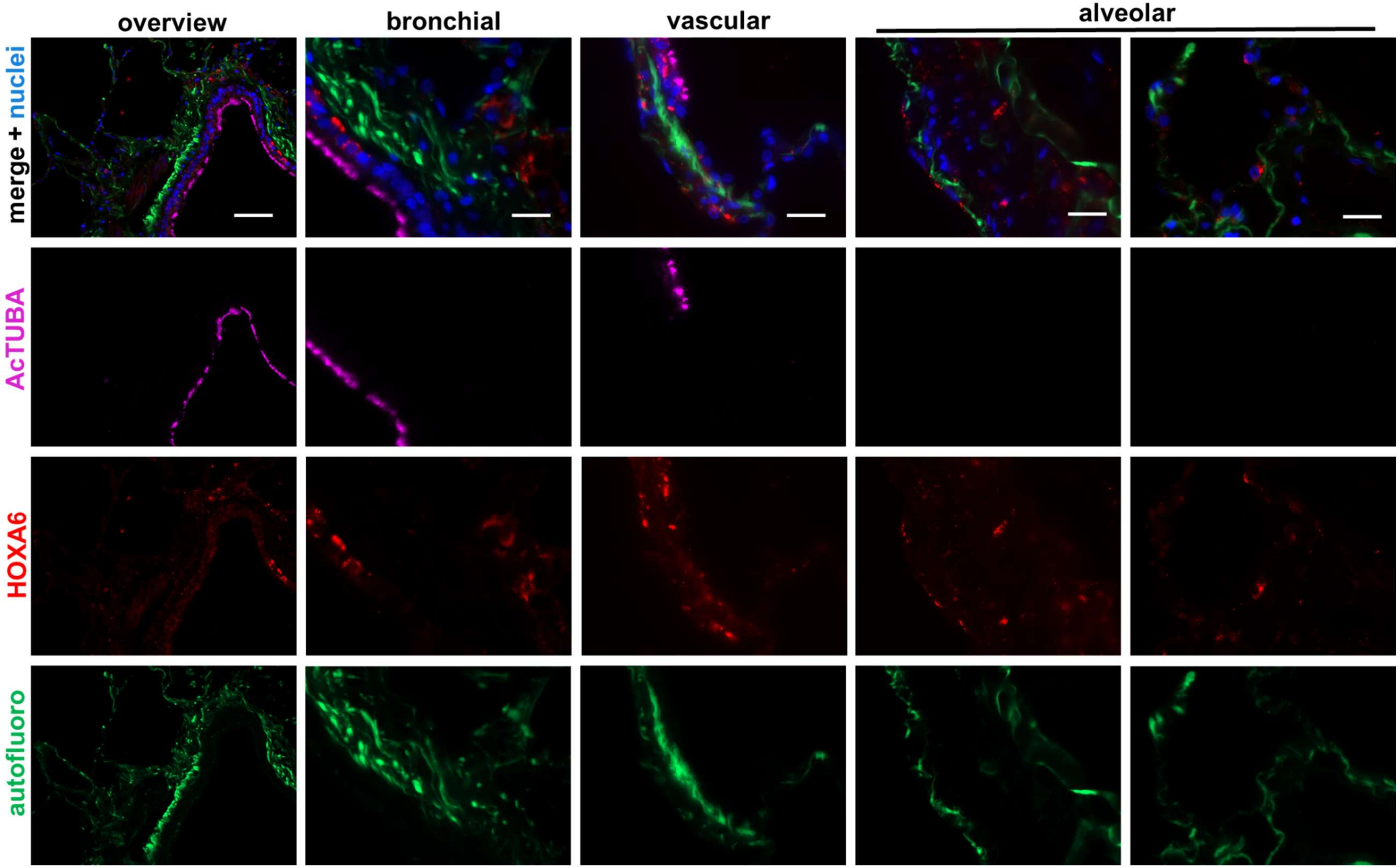

Immunofluorescent analysis of HOXA6 (I). Double-immunofluorescent staining of normal lung sections was performed using antibodies against HOXA6 (red) together with acetylated tubulin A (AcTUBA; violet). Nuclei were visualized in blue. Single channel images of the merged pictures depicted in Figure 4 are shown. Scale bar indicate 100 µm (overview) and 10µm (higher magnifications).

Supplemental Figure S12

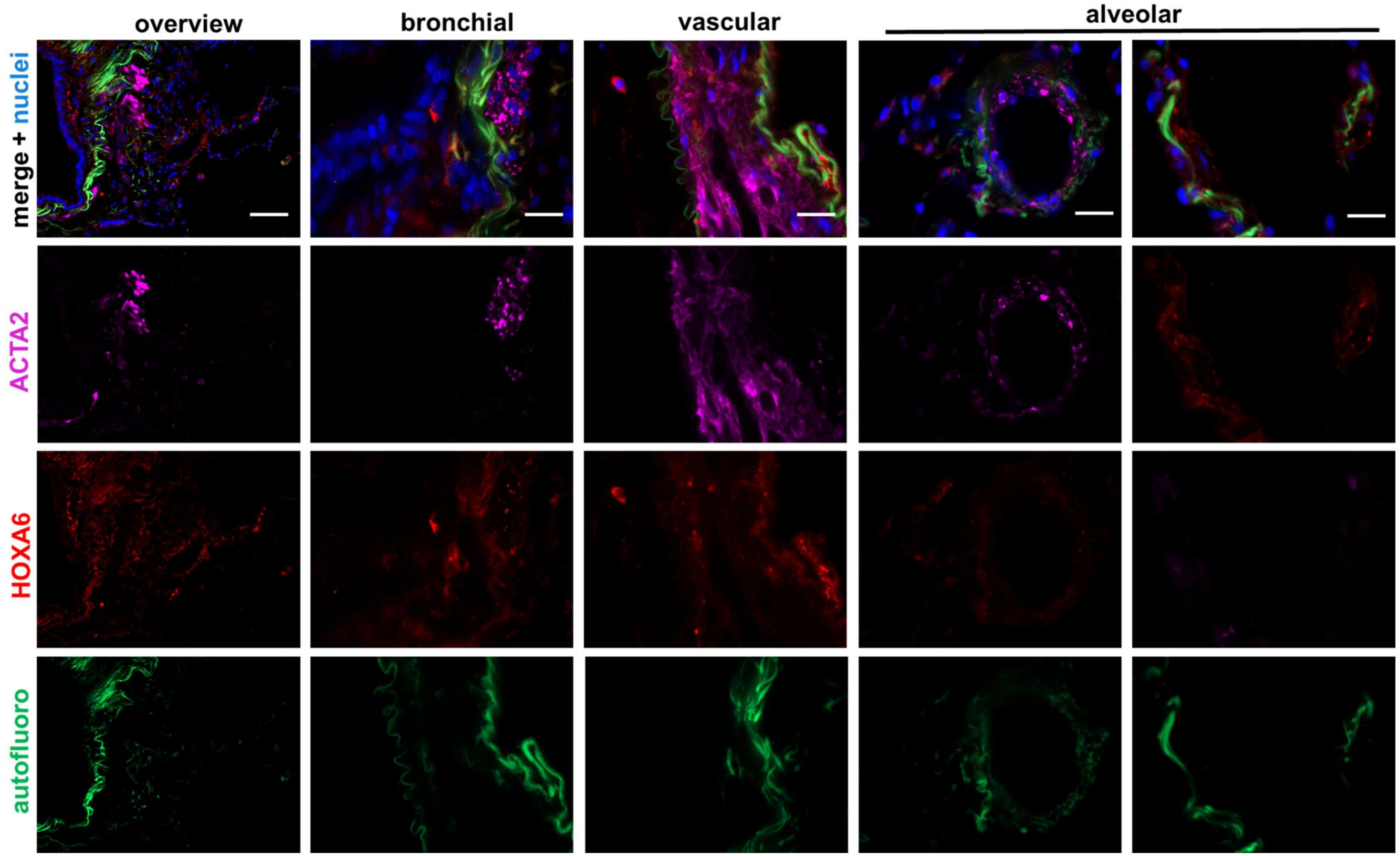

Immunofluorescent analysis of HOXA6 (II). Double-immunofluorescent staining of normal lung sections was performed using antibodies against HOXA6 (red) and ACTA2 (violet). Nuclei were visualized in blue. Single channel images of the merged pictures depicted in Figure 4 are shown. Scale bar indicate 100 µm (overview) and 10µm (higher magnifications).

Supplemental Figure S13

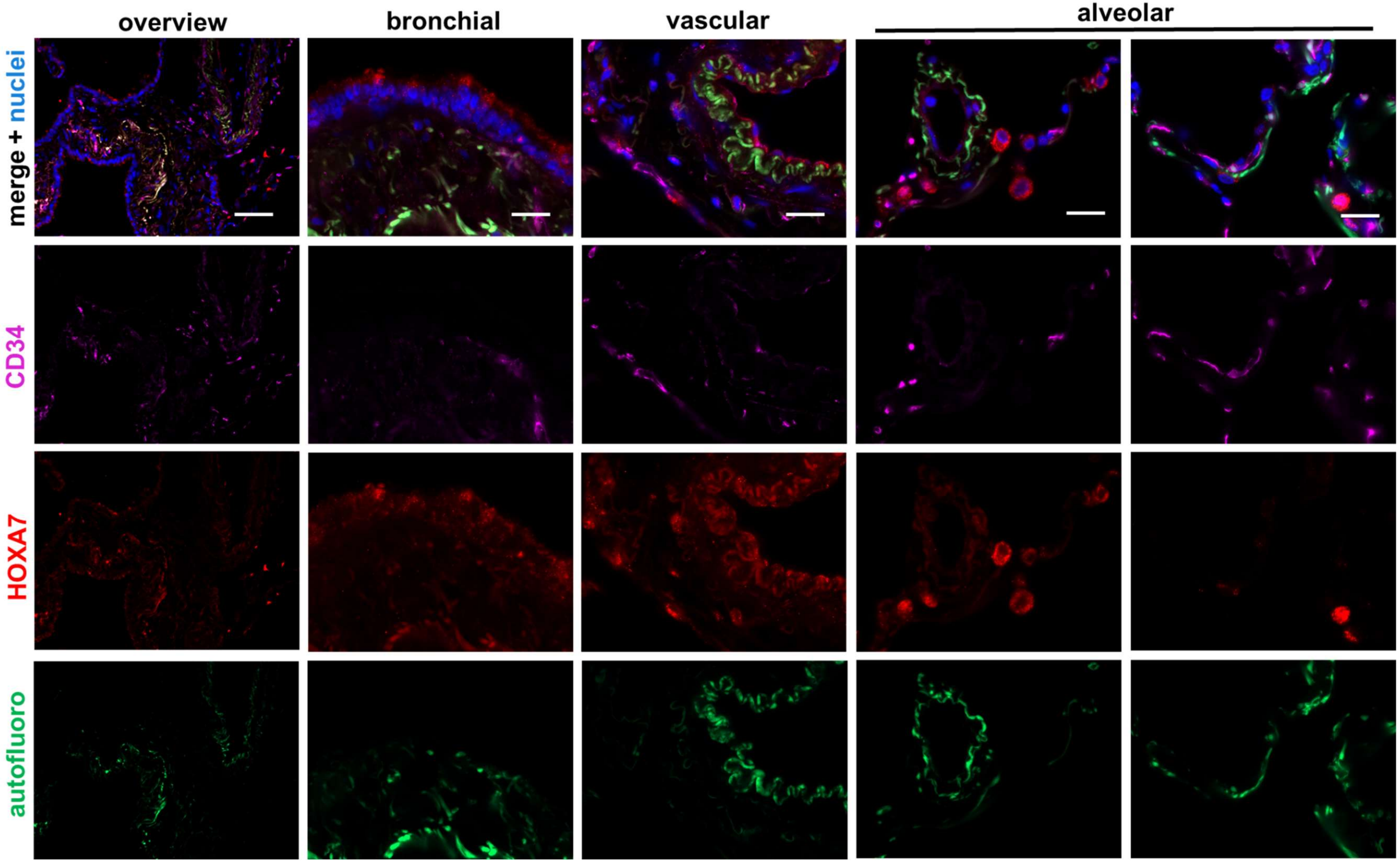

Immunofluorescent analysis of HOXA7 (I). Double-immunofluorescent staining of normal lung sections was performed using antibodies against HOXA7 (red) and CD34 (violet). Nuclei were visualized in blue. Single channel images of the merged pictures depicted in Figure 4 are shown. Scale bar indicate 100 µm (overview) and 10µm (higher magnifications).

Supplemental Figure S14

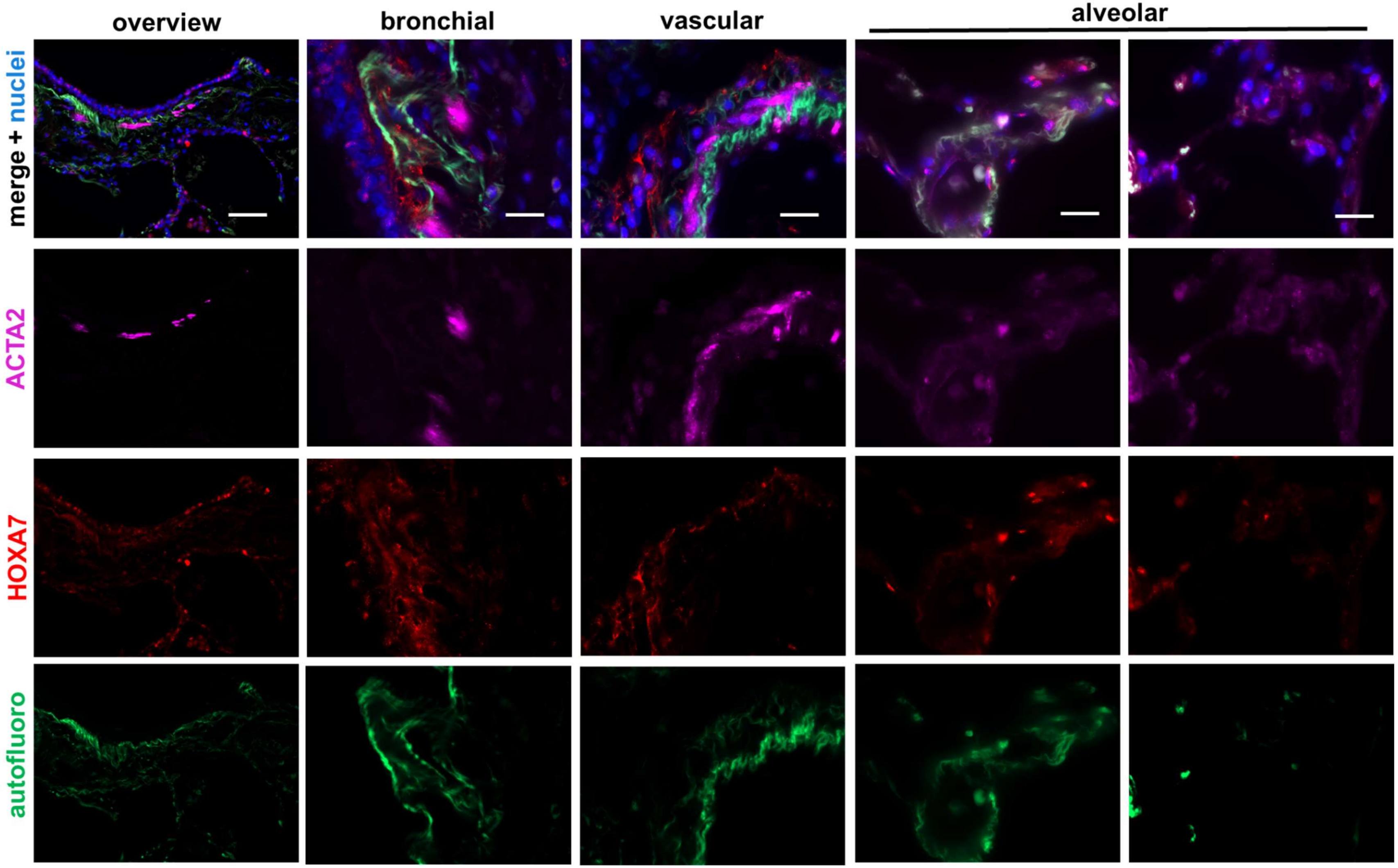

Immunofluorescent analysis of HOXA7 (II). Double-immunofluorescent staining of normal lung sections was performed using antibodies against HOXA7 (red) and ACTA2 (violet). Nuclei were visualized in blue. Single channel images of the merged pictures depicted in Figure 4 are shown. Scale bar indicate 100 µm (overview) and 10µm (higher magnifications).

Supplemental Figure S15

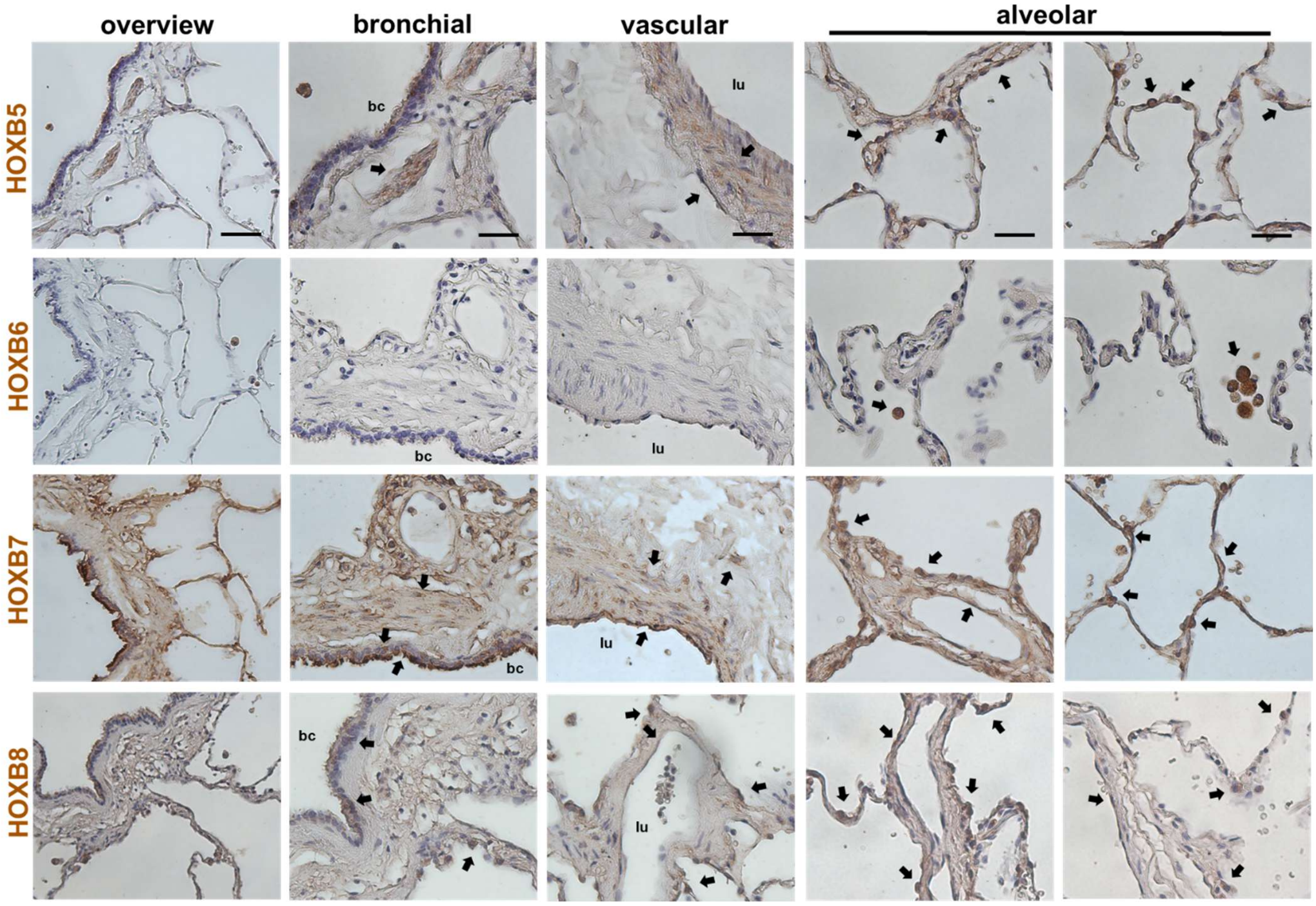

Immunohistochemical analysis of HOXB proteins. Immunohistochemical staining of normal lung tissue sections was performed using the indicated HOXB antibodies and DAB staining (brown). Nuclei were counterstained with hematoxylin (blue). Representative lung photographs of bronchial, vascular and alveolar structures are shown (magnifications). Arrows highlight immunoreactive structures. bc bronchial epithelium, lu lumen. Scale bar indicates 100 μm (left panel) and 10 μm (higher magnification images).

Supplemental Figure S16

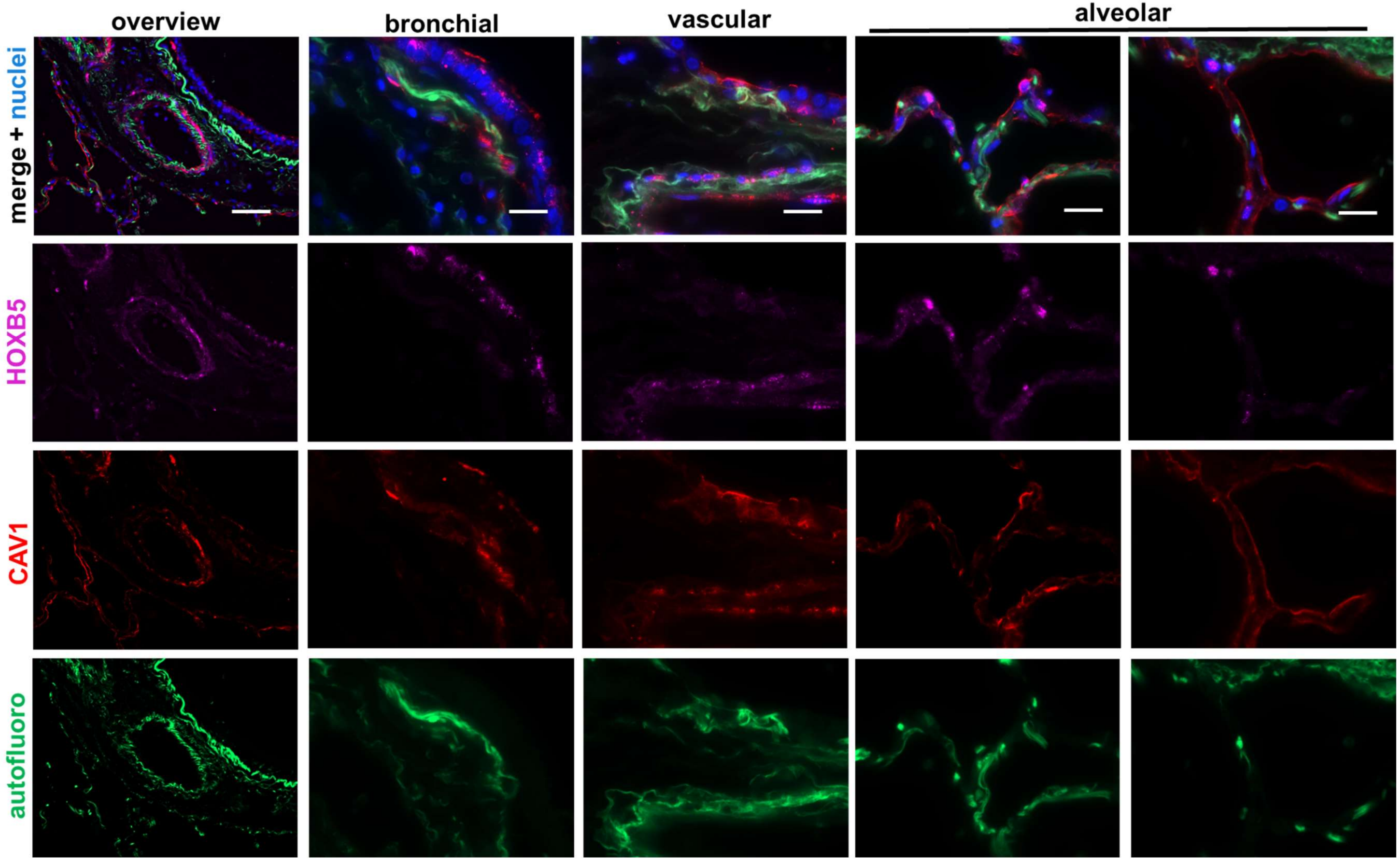

Immunofluorescent analysis of HOXB5. Double-immunofluorescent staining of normal lung sections was performed using antibodies against CAV1 (red) and HOXB5 (violet). Nuclei were visualized in blue. Single channel images of the merged pictures depicted in Figure 5 are shown. Scale bar indicate 100 µm (overview) and 10µm (higher magnifications).

Supplemental Figure S17

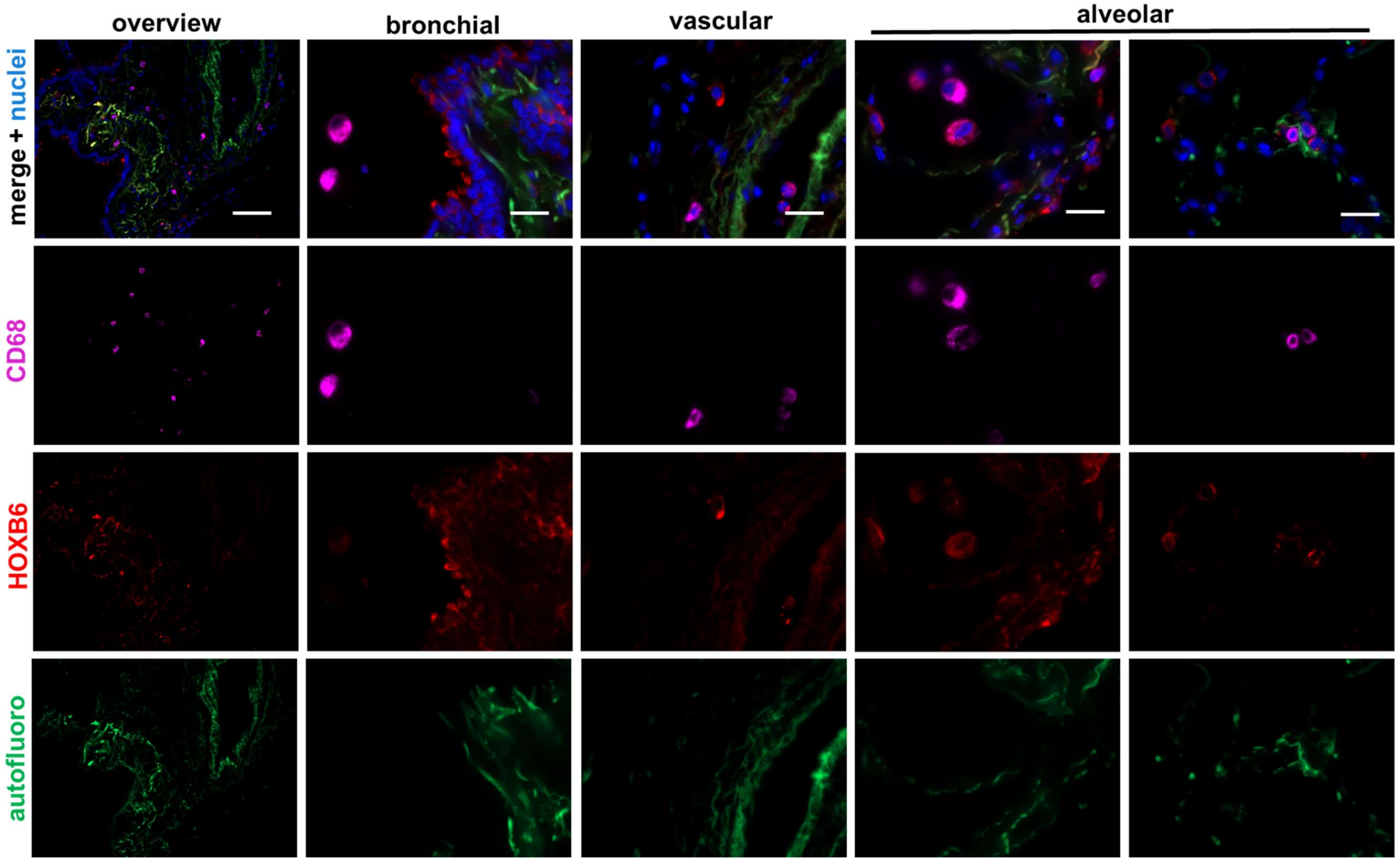

Immunofluorescent analysis of HOXB6. Double-immunofluorescent staining of normal lung sections was performed using antibodies against HOXB6 (red) and CD68 (violet). Nuclei were visualized in blue. Single channel images of the merged pictures depicted in Figure 5 are shown. Scale bar indicate 100 µm (overview) and 10µm (higher magnifications).

Supplemental Figure S18

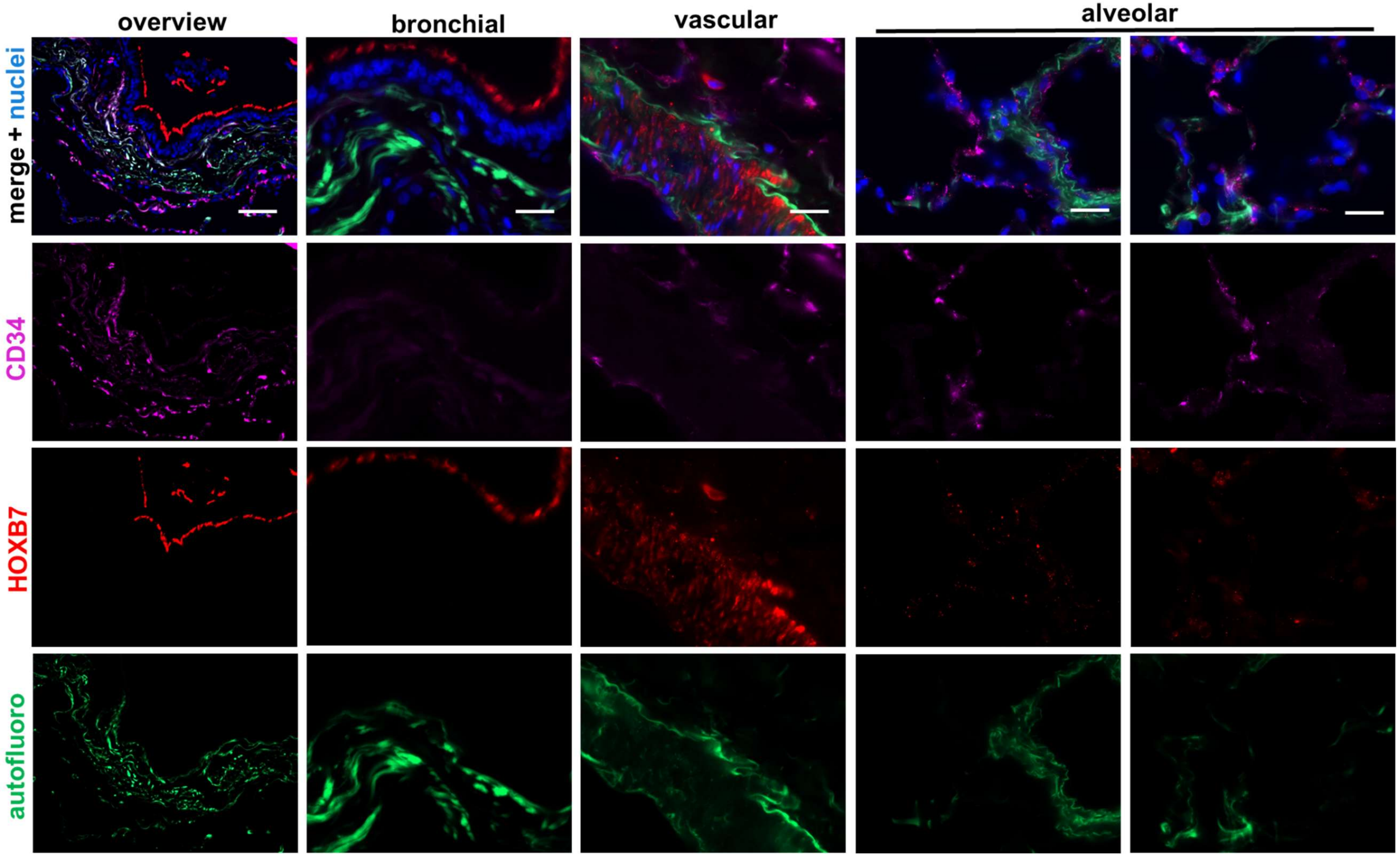

Immunofluorescent analysis of HOXB7. Double-immunofluorescent staining of normal lung sections was performed using antibodies against HOXB7 (red) and CD34 (violet). Nuclei were visualized in blue. Single channel images of the merged pictures depicted in Figure 5 are shown. Scale bar indicate 100 µm (overview) and 10µm (higher magnifications).

Supplemental Figure S19

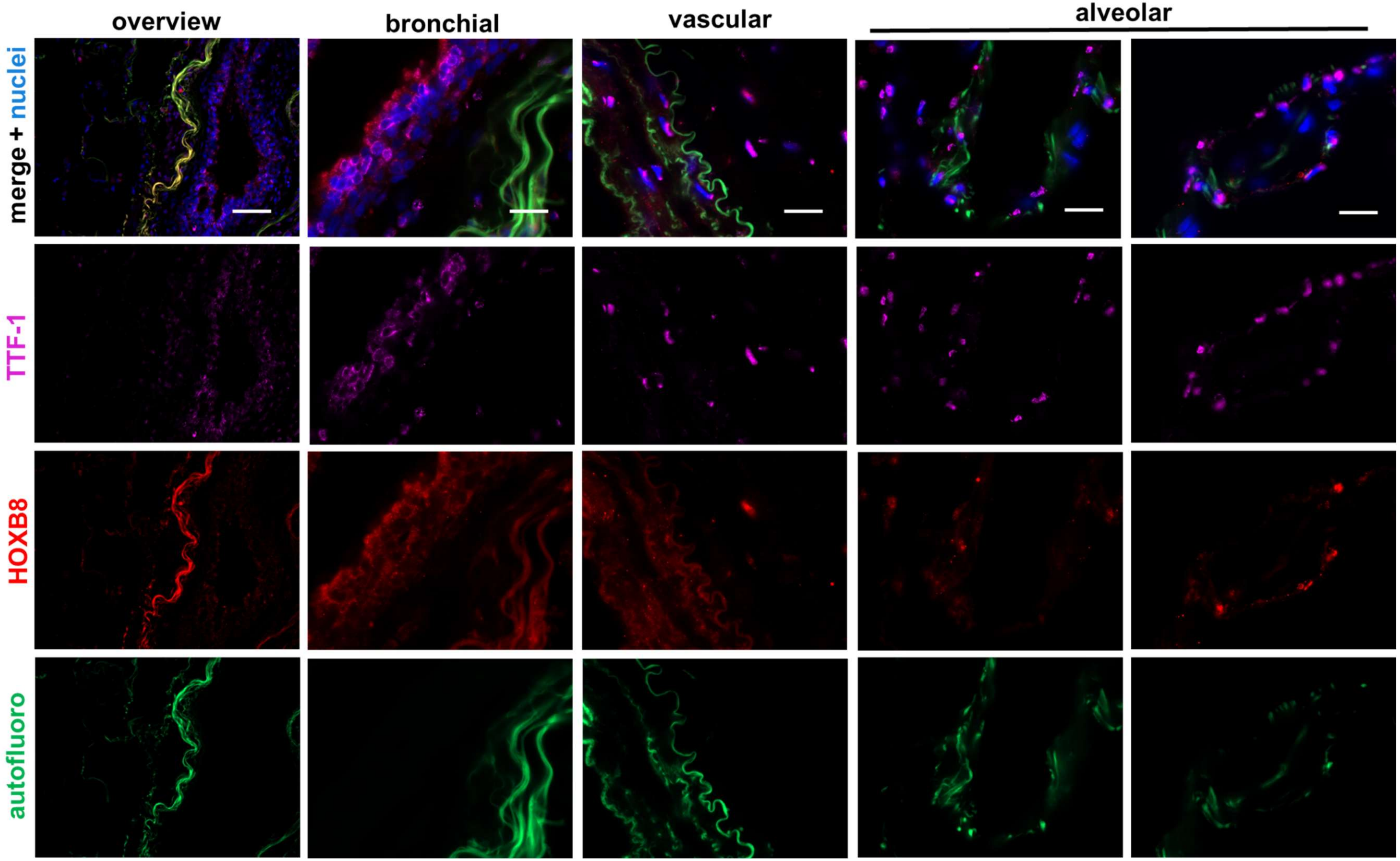

Immunofluorescent analysis of HOXB8. Double-immunofluorescent staining of normal lung sections was performed using antibodies against HOXA7 (red) and TTF-1 (violet). Nuclei were visualized in blue. Single channel images of the merged pictures depicted in Figure 5 are shown. Scale bar indicate 100 µm (overview) and 10µm (higher magnifications).

Supplemental Figure S20

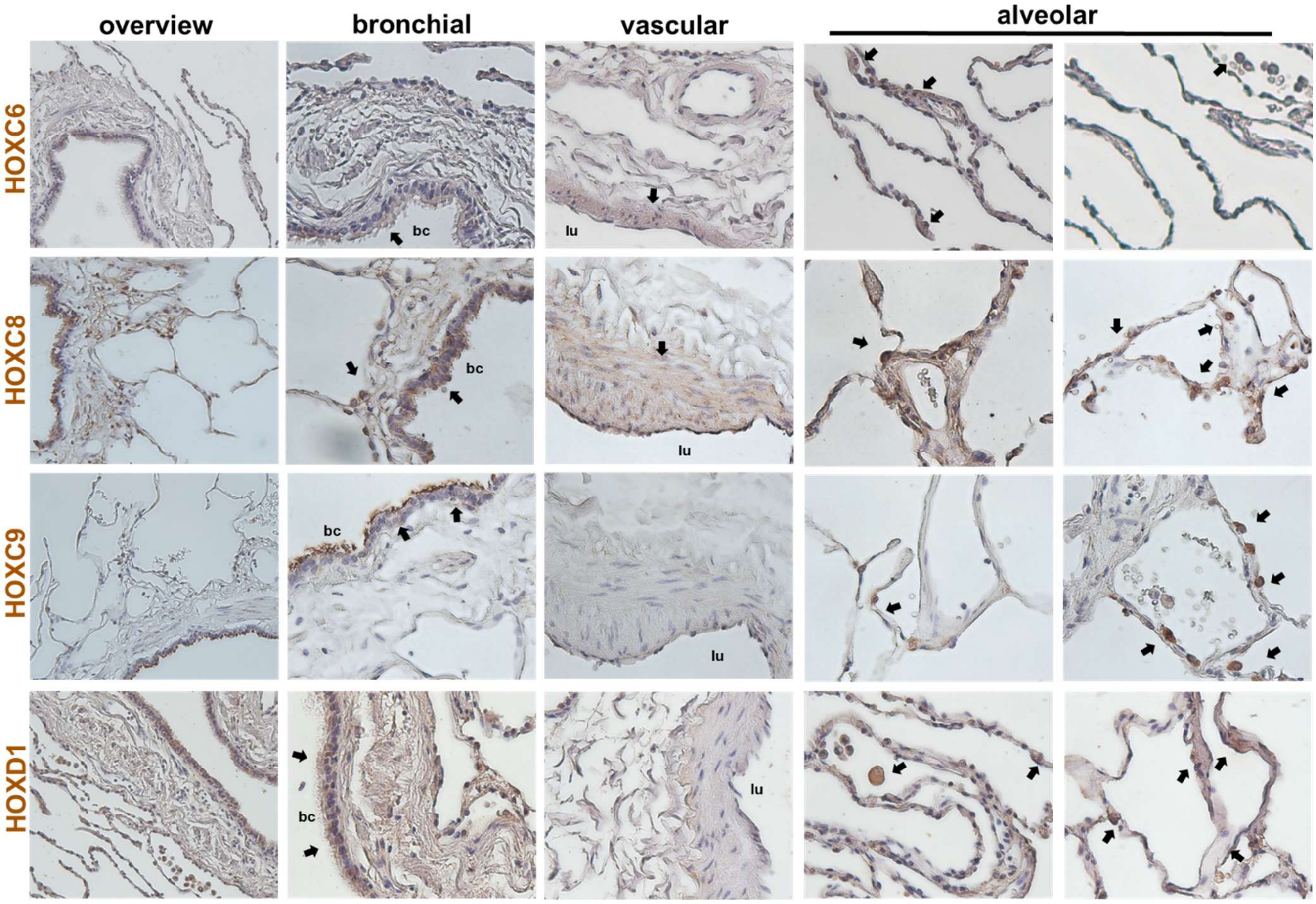

Immunohistochemical analysis of HOXC proteins and HOXD1. Immunohistochemical staining of normal lung tissue sections was performed using the indicated HOXC antibodies as well as HOXD1 and DAB staining (brown). Nuclei were counterstained with hematoxylin (blue). Representative lung photographs of bronchial, vascular and alveolar structures are shown (magnifications). Arrows highlight immunoreactive structures. bc bronchial epithelium, lu lumen. Scale bar indicates 100 µm (left panel) and 10 µm (higher magnification images).

Supplemental Figure S21

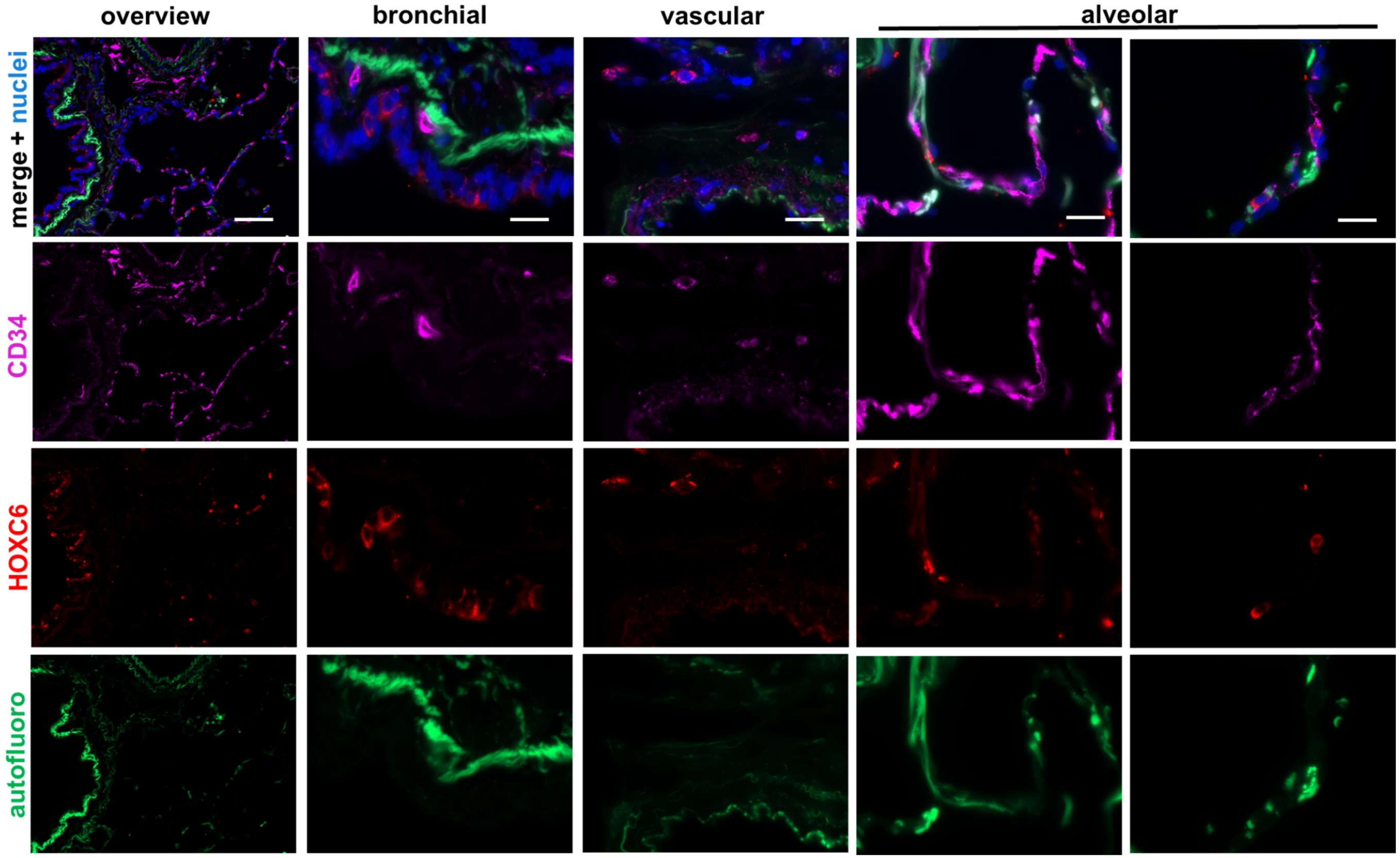

Immunofluorescent analysis of HOXC6. Double-immunofluorescent staining of normal lung sections was performed using antibodies against HOXC6 (red) and CD34 (violet). Nuclei were visualized in blue. Single channel images of the merged pictures depicted in Figure 6 are shown. Scale bar indicate 100 µm (overview) and 10µm (higher magnifications).

Supplemental Figure S22

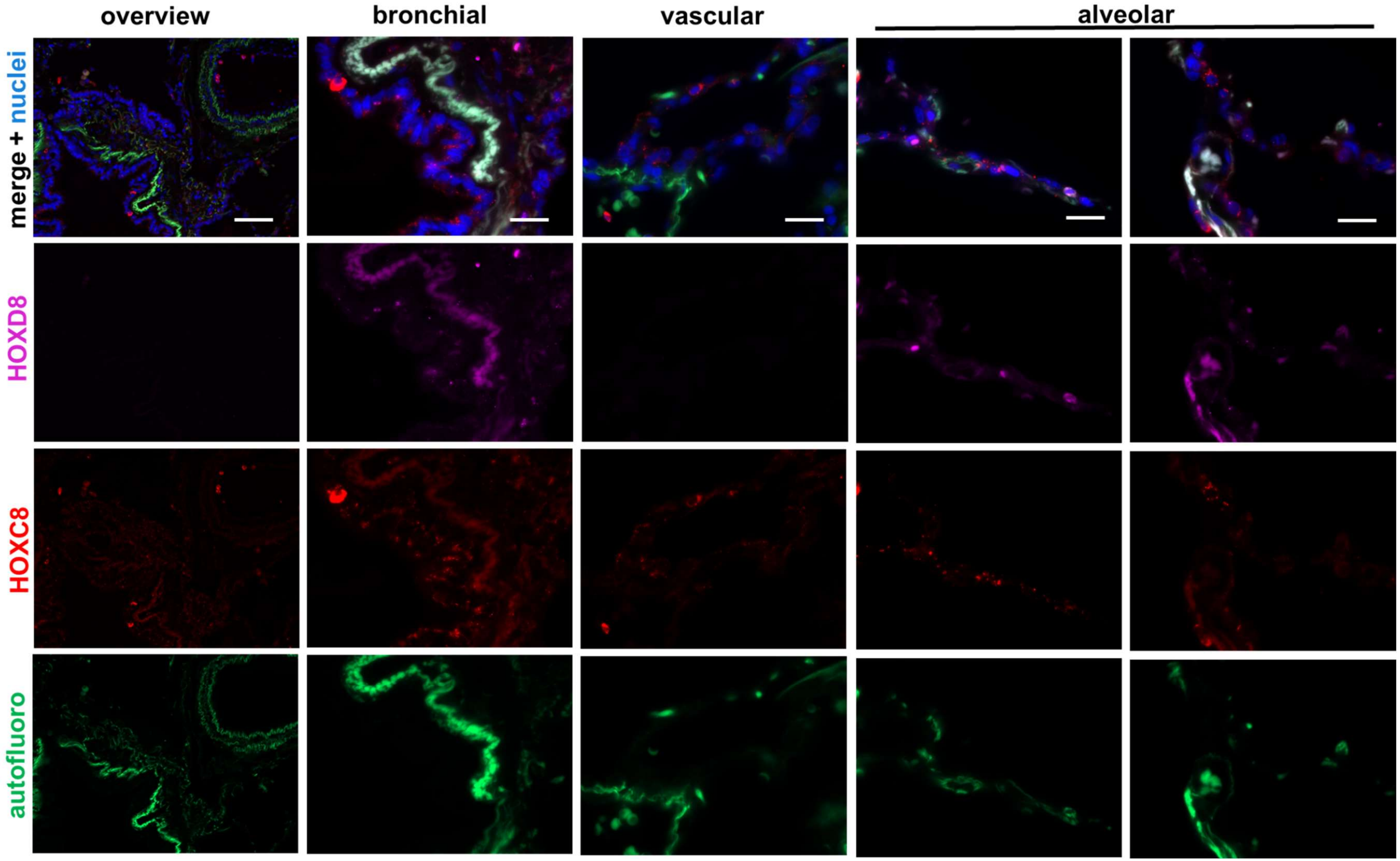

Immunofluorescent analysis of HOXC8 and HOXD8. Double-immunofluorescent staining of normal lung sections was performed using antibodies against HOXC8 (red) and HOXD8 (violet). Nuclei were visualized in blue. Single channel images of the merged pictures depicted in Figure 6 are shown. Scale bar indicate 100 µm (overview) and 10µm (higher magnifications).

Supplemental Figure S23

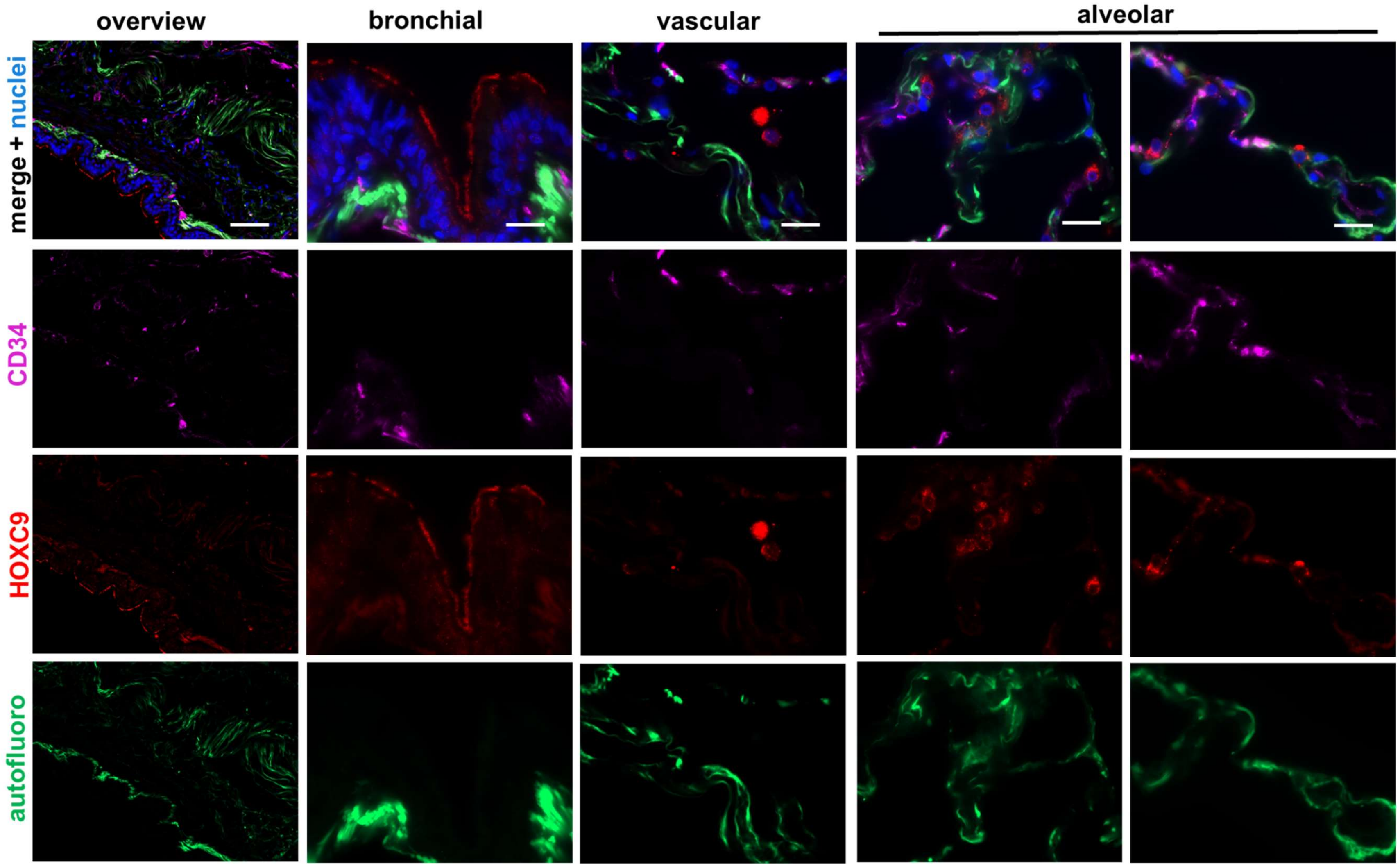

Immunofluorescent analysis of HOXC9 (I). Double-immunofluorescent staining of normal lung sections was performed using antibodies against HOXC9 (red) and CD34 (violet). Nuclei were visualized in blue. Single channel images of the merged pictures depicted in Figure 6 are shown. Scale bar indicate 100 µm (overview) and 10µm (higher magnifications).

Supplemental Figure S24

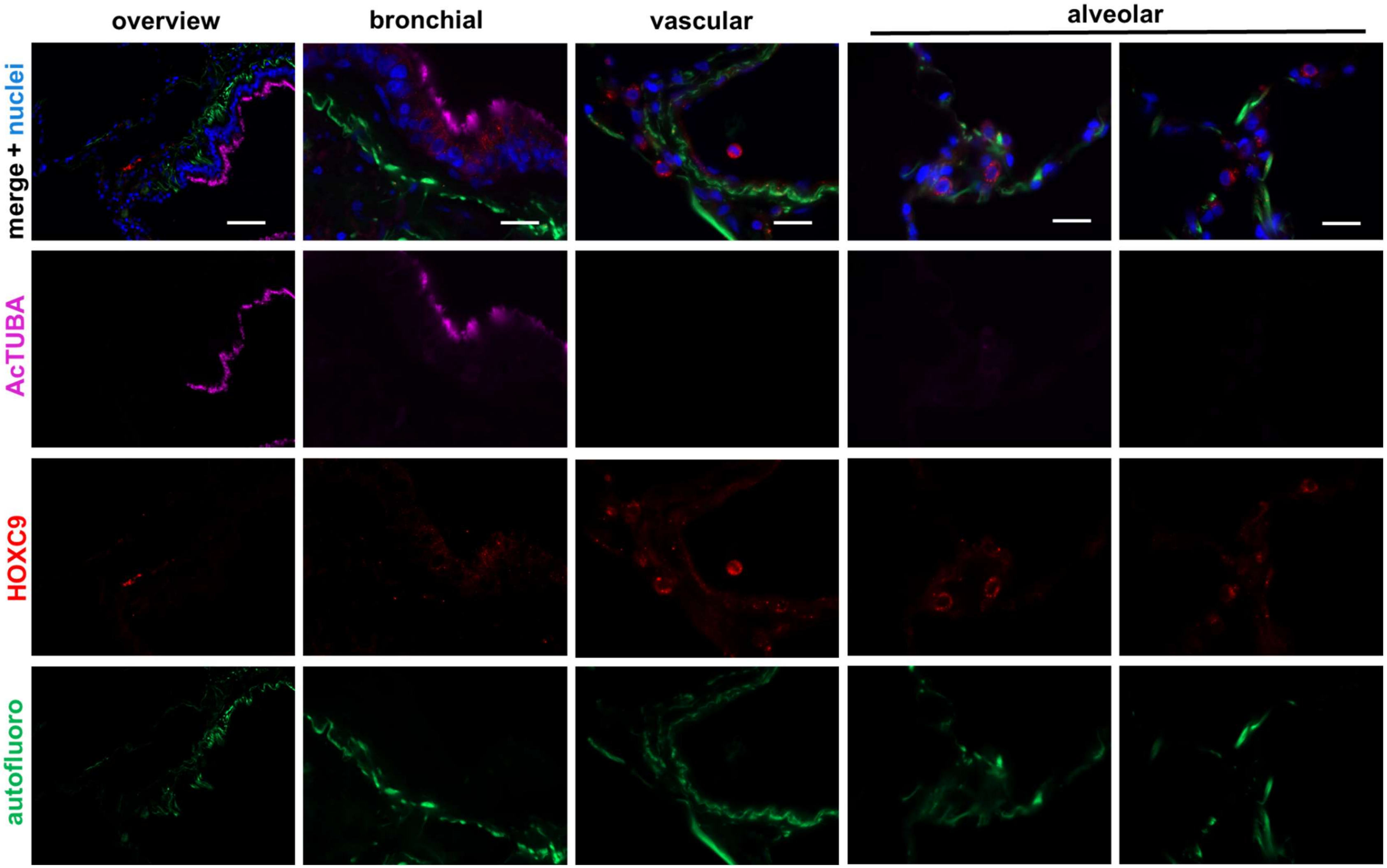

Immunofluorescent analysis of HOXC9 (II). Double-immunofluorescent staining of normal lung sections was performed using antibodies against C9 (red) and AcTUBA(violet). Nuclei were visualized in blue. Single channel images of the merged pictures depicted in Figure 6 are shown. Scale bar indicate 100 µm (overview) and 10µm (higher magnifications).

Supplemental Figure S25

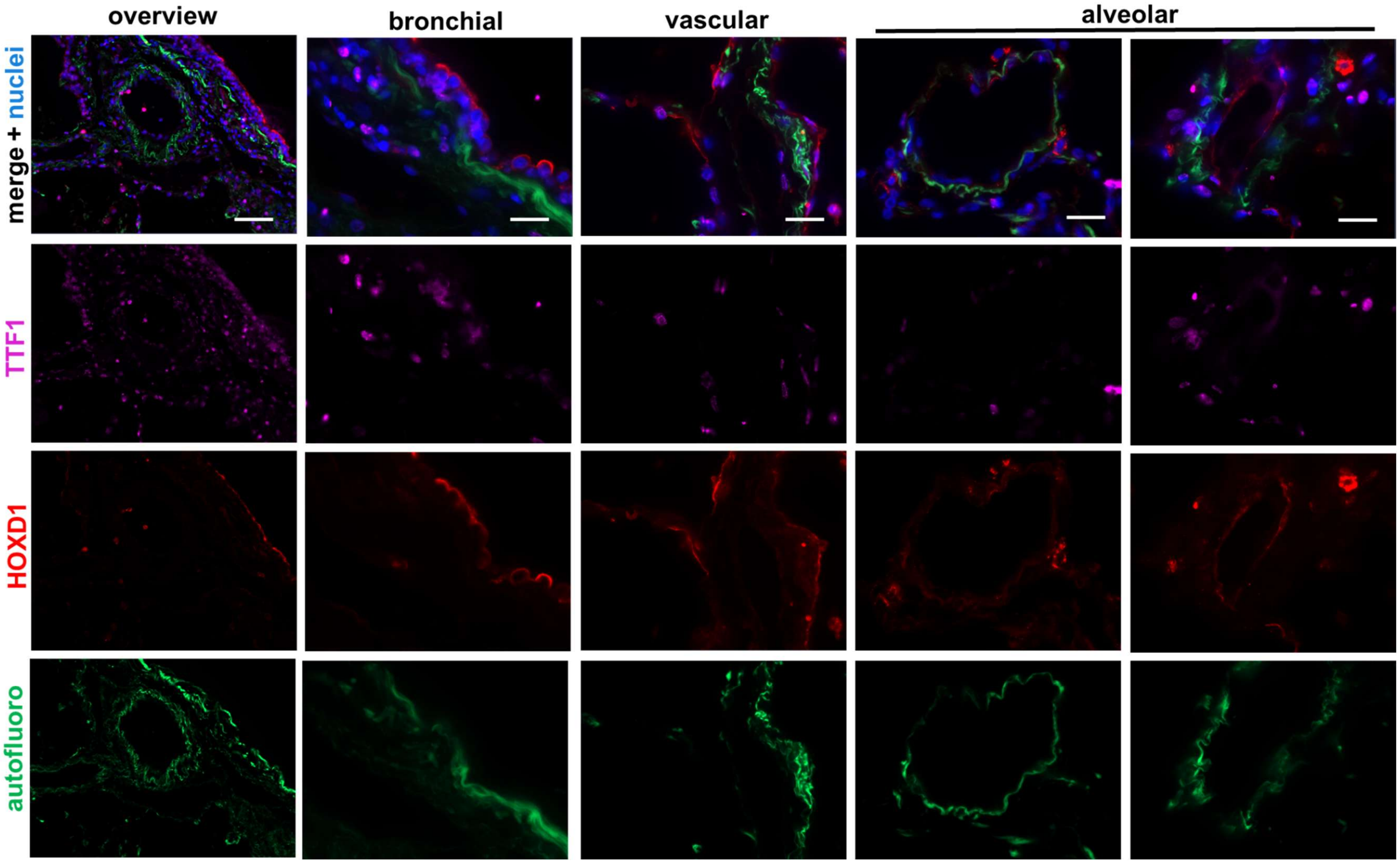

Immunofluorescent analysis of HOXD1 (I). Double-immunofluorescent staining of normal lung sections was performed using antibodies against HOXD1 (red) and TTF-1 (violet). Nuclei were visualized in blue. Single channel images of the merged pictures depicted in Figure 6 are shown. Scale bar indicate 100 µm (overview) and 10µm (higher magnifications).

Supplemental Figure S26

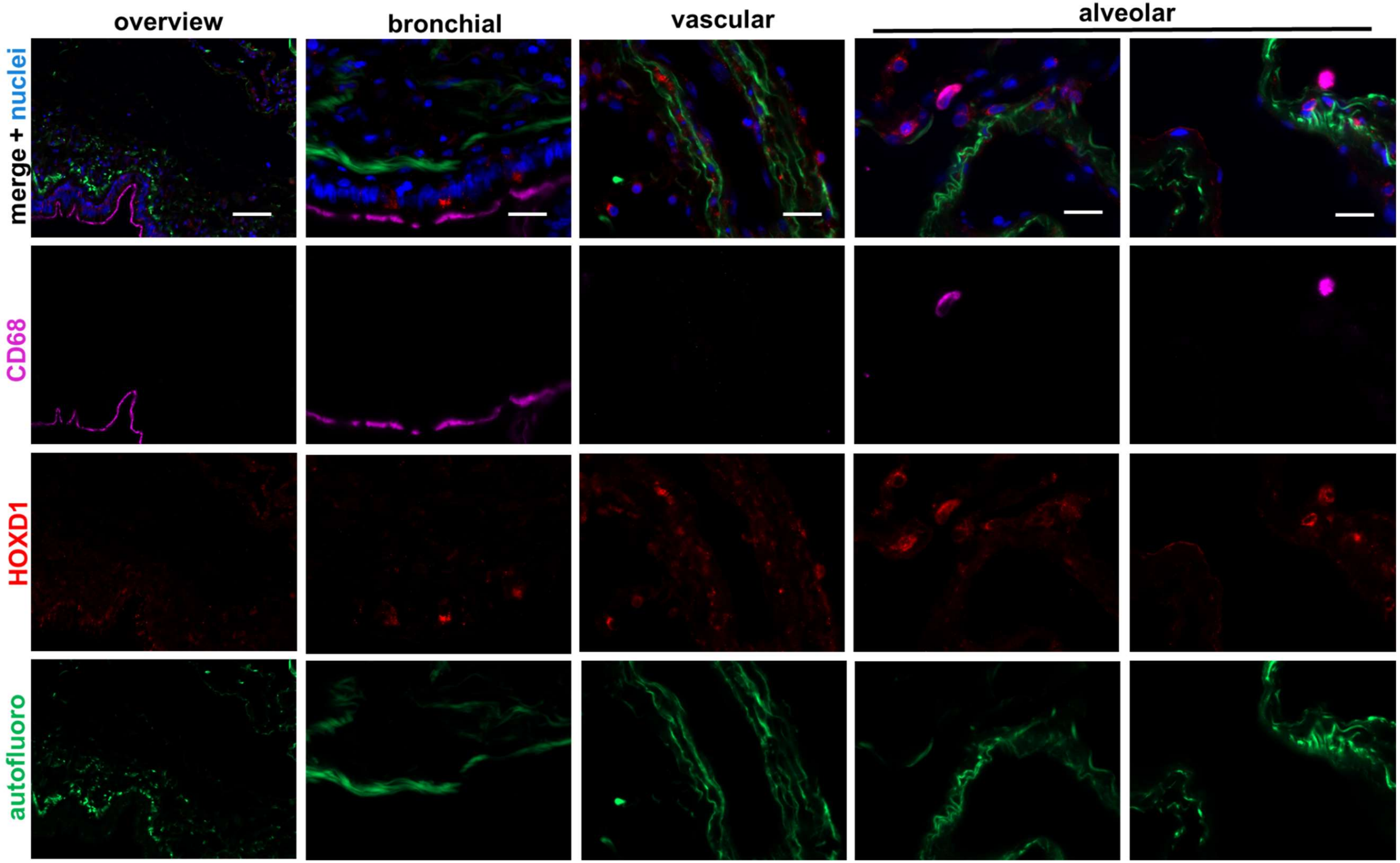

Immunofluorescent analysis of HOXD1 (II). Double-immunofluorescent staining of normal lung sections was performed using antibodies against HOXD1 (red) and CD68 (violet). Nuclei were visualized in blue. Single channel images of the merged pictures depicted in Figure 6 are shown. Scale bar indicate 100 µm (overview) and 10µm (higher magnifications).

Supplemental Figure S27

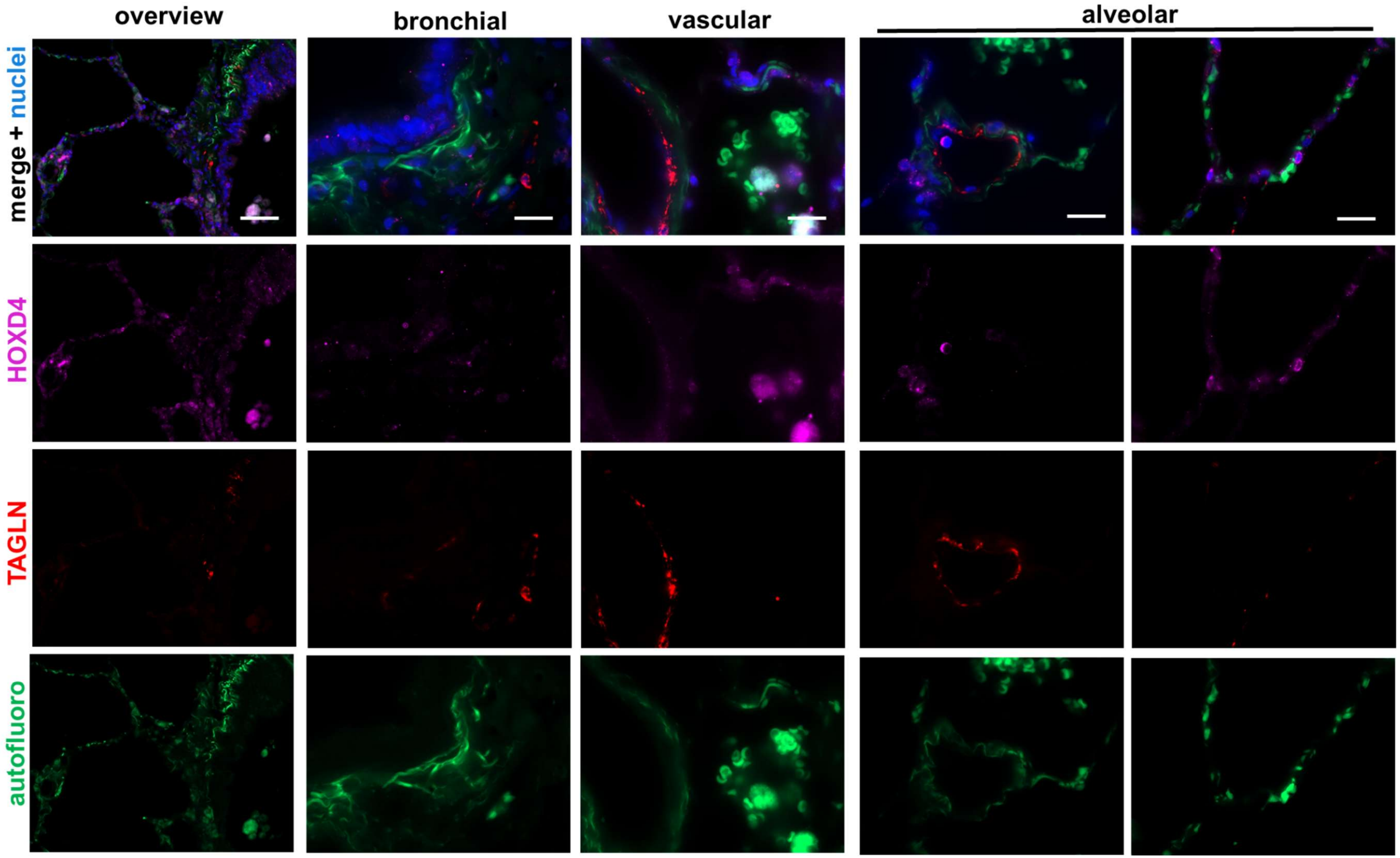

Immunofluorescent analysis of HOXD4. Double-immunofluorescent staining of normal lung sections was performed using antibodies against Tagln (red) and HOXD4 (violet). Nuclei were visualized in blue. Single channel images of the merged pictures depicted in Figure 6 are shown. Scale bar indicate 100 µm (overview) and 10µm (higher magnifications).

Supplemental Figure S28

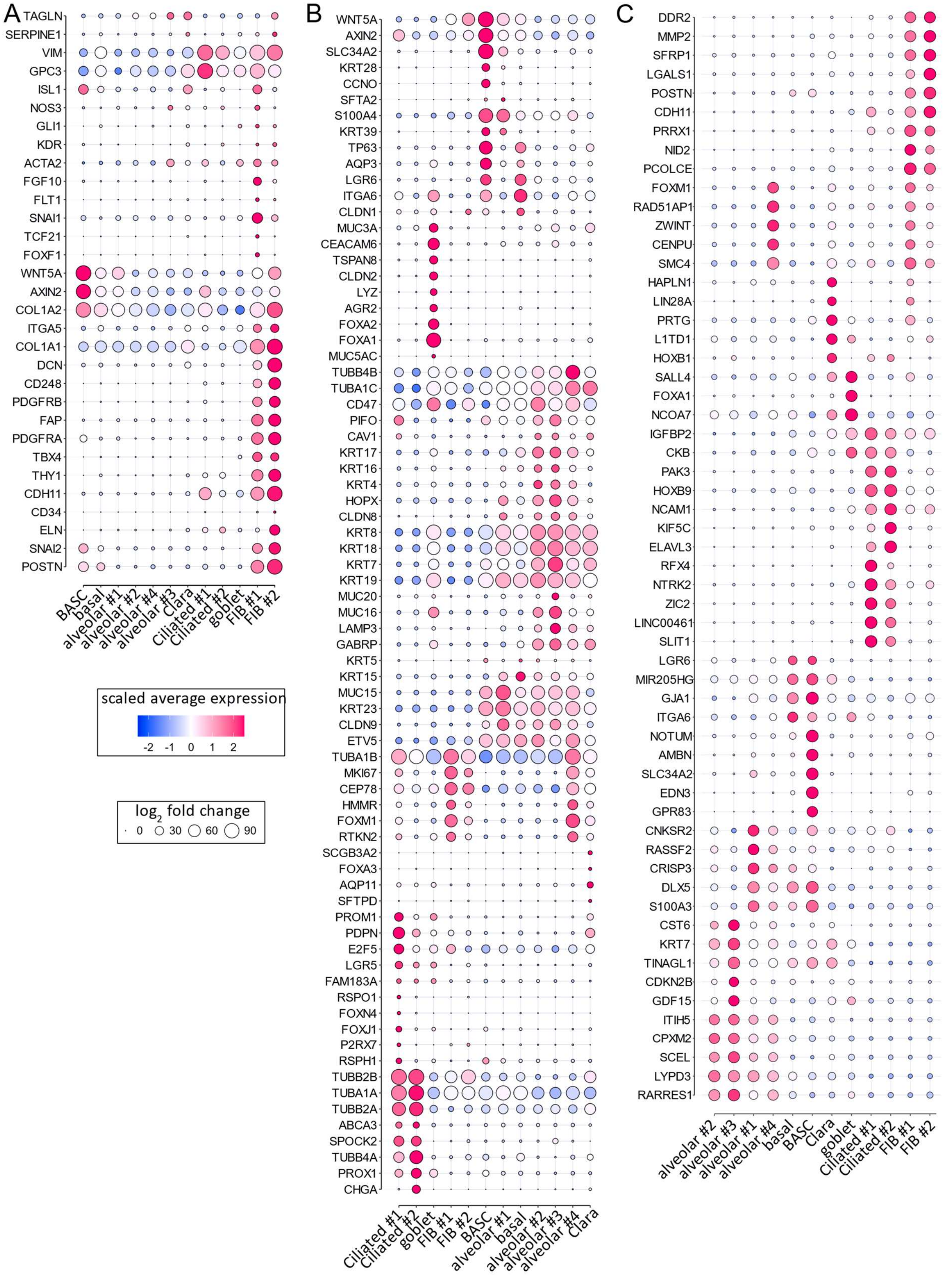

Single cell analysis of the composition of lung organoids. (A) Dot plot of indicated mesenchymal marker genes. (B) Dot plot of epithelial marker genes. (C) Dot plot of the top five differentially expressed genes per cluster. Size of the dots indicate the percentage of cells in which this gene was found, the color indicates the normalized value of the expression.

Supplemental Figure S29

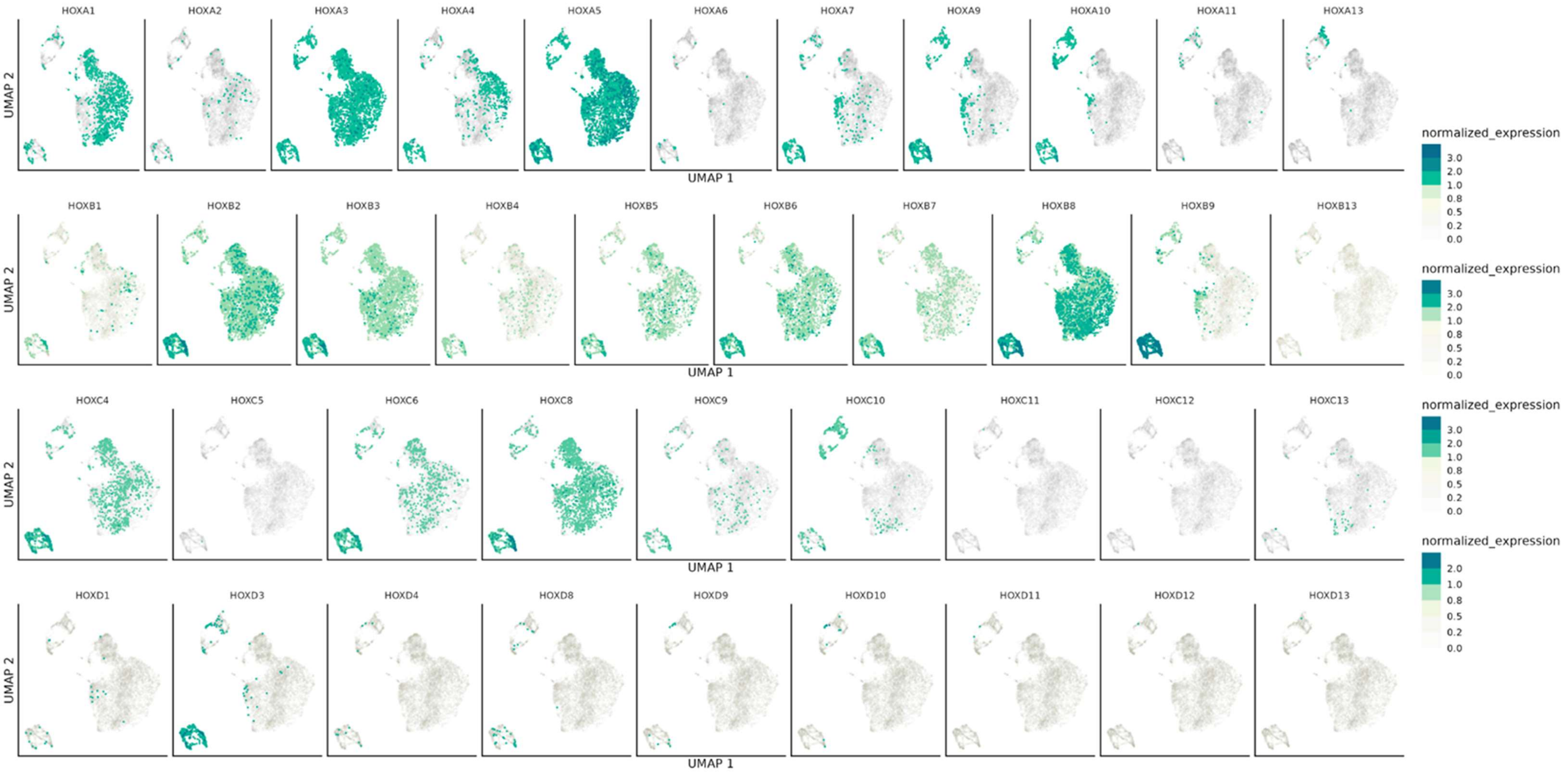

Cell-type dependent HOX expressions in lung organoids. Expression map (UMAP, 12 clusters) of the 39 human HOX genes in all clusters.

Supplemental Figure S30

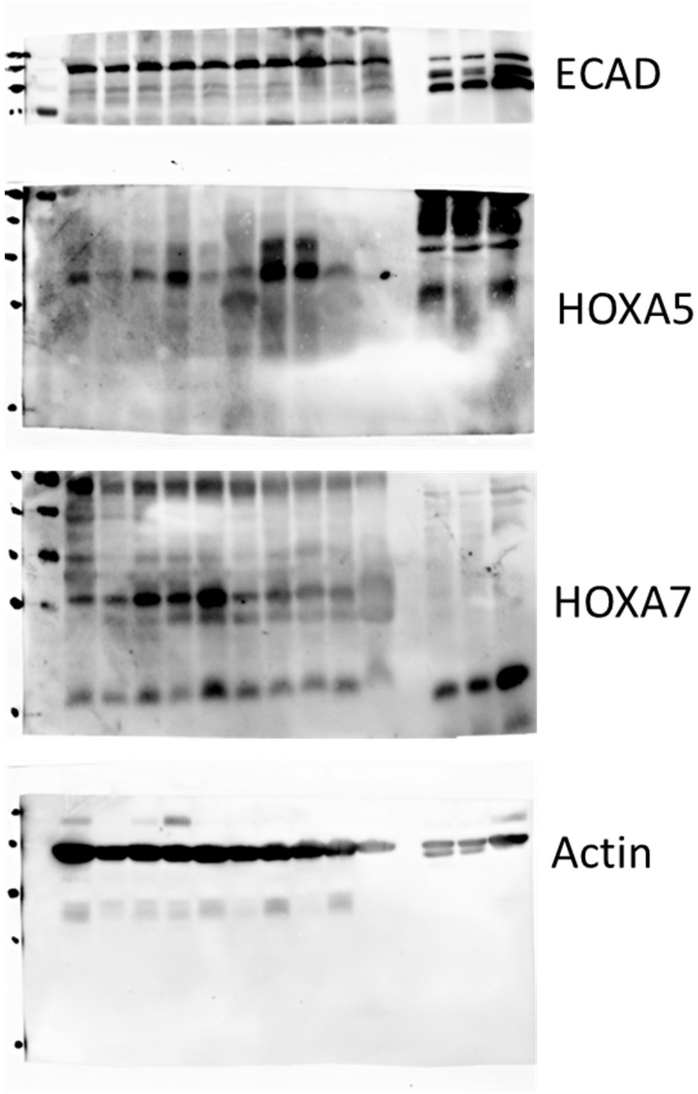

Uncropped gels as depicted in Supplemental Figure S1B.
